# Photonic Applications of Betanin

**DOI:** 10.3390/molecules31183155

**Published:** 2026-09-08

**Authors:** Pierre D. Harvey

**Affiliations:** Département de Chimie, Faculté des Sciences, Université de Sherbrooke, Sherbrooke, QC J1K 2R1, Canada; pierre.harvey@usherbrooke.ca

**Keywords:** betanin, betalains, betacyanins, fluorescence, organic light emitting diodes, dye-sensitized solar cells, nonlinear optical materials, nanoparticles, singlet–singlet energy transfer, photo-induced electron transfer

## Abstract

Betanin is the main betacyanin and major pigment extracted from red beets (*Beta vulgaris*). While its applications as food colorant, color-based sensors, antioxidant, anti-inflammatory, and antimicrobial traits are well-known, this natural dye is underestimated and underexploited in the field of light- and electric-responsive materials and devices. This comprehensive review describes the optical properties and molecular orbitals of this chromophore, along with its excited-state relaxation dynamics and emission quantum yields. Other relevant and basic behaviors of its excited-state features, such as *cis-trans*-photoisomerization, singlet–singlet energy transfer, photo-induced electron transfer, photosensitization of ^1^O_2_, and relative photo-instability are also presented in some relevant details. Photoconductive materials based on betanin and their related devices such as organic light emitting diodes, and dye-sensitized solar cells, are also surveyed. Other visible light-driven processes such as photodynamic processes and heterogeneous catalysis based on betanin-containing assembly/composites are demonstrated. Finally, the use of betanin as nonlinear optical materials is addressed.

## 1. Introduction

**Figure 1:**
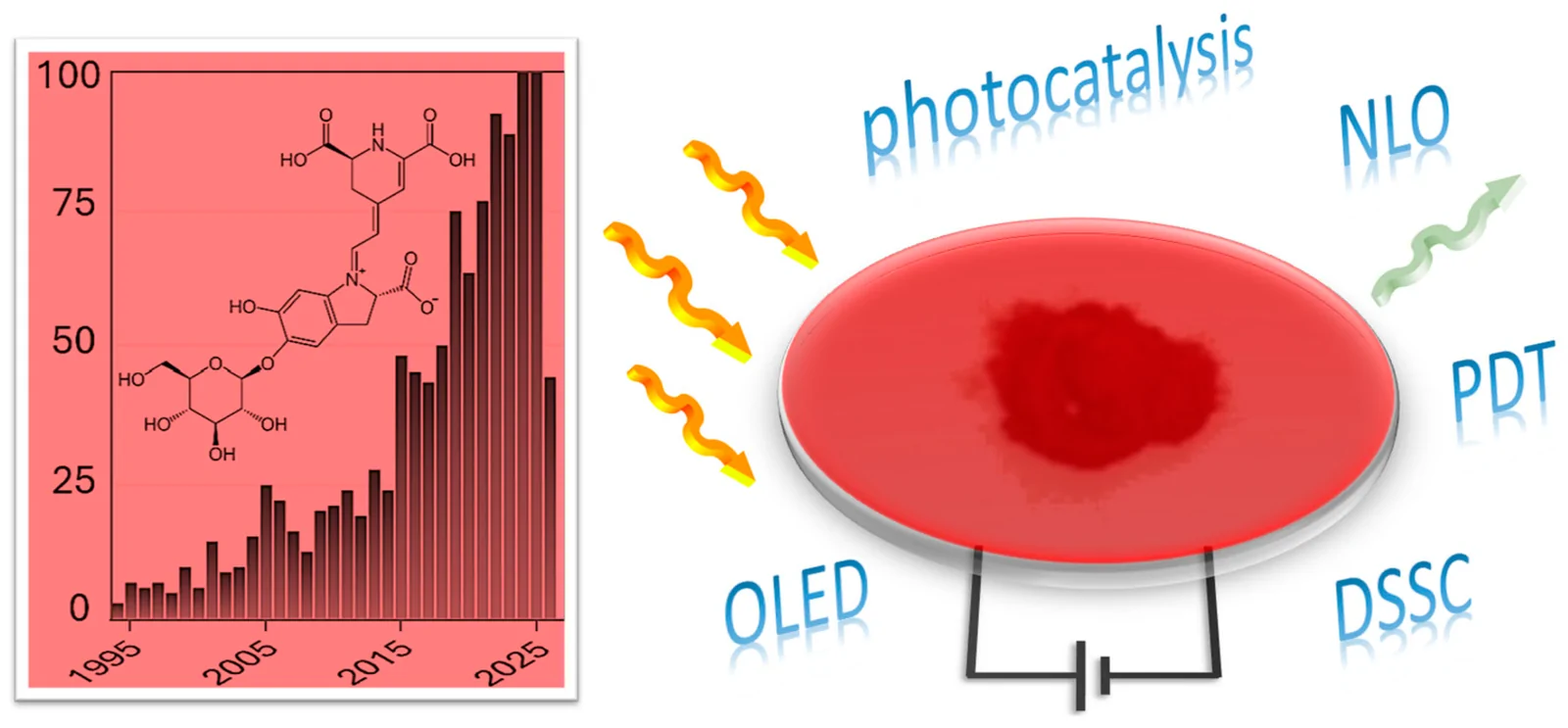
**Left**: Evolution of the number of research articles devoted to betanin over the past three decades according to the search engine Scifinder. This search has been performed on 3 July 2026 and excludes patents, clinical trials, and commentaries. **Right**: Applications resulting from betanin as a pigment that were reported over the past 15 years.

The zwitterionic betanin molecule (betanidin-5-O-β-glucoside) belongs to the family of betacyanins and is the major pigment of Betalains, which are mainly extracted from red beets (*Beta vulgaris*). The betacyanin family is structurally characterized by the presence of a chemically condensed betalamic acid (responsible for the color) with (2S)-5,6-dihydroxy-2,3-dihydro-1H-indole-2-carboxylic acid (cyclo-DOPA), which modulates the photophysical properties. So, the reddish-violet coloration of betanin confers its use as a food colorant but has gained increasing research interest over the past several decades for many other purposes (Figure 1). Among a multitude features, it also exhibits rich beneficial antioxidant and therapeutic properties such as anticancer, antidiabetic, antimicrobial and neuroprotective activities just to state a few [1,2,3,4]. In nature, this pigment is also produced by some plants to render their flowers attractive to pollinators by optically tuning the emitted wavelength perceived by these insects [5,6]. Large scale utilization of betanin mainly concerns non-toxic food and beverage colorants [7,8], and sometimes in cosmetics [9]. However, its main drawback is its thermal and chemical stability [10], so storage or long-term applications are rather limited or cautiously selected.

Betanin is also a photosensitizer of singlet oxygen, ^1^O_2_, with potential applications in dentistry (sterilization of root canals; patent WO2016106173 A1 30 June 2016) and photodynamic therapy (PDT) [11]. Ironically, betanin also reacts efficiently with ^1^O_2_ to form 2-decarboxy-2,3-dehydrobetanin with a bimolecular (diffusion limited) rate of 1.20 ± 0.15 × 10^8^ M^−1^s^−1^ in D_2_O [12] and was even used as a chemical probe to evaluate the photosensitization quantum yield (Φ_Δ_) of ^1^O_2_, for which the rate of reaction is also found to be proportional to the irradiation lamp intensity [13]. While the photochemical instability of betanin at 25 °C in the presence of molecular oxygen in solution has been known for quite some time [14], this reactivity has been recently and cleverly exploited to protect ARPE-19 cells (retinal pigment epithelial cells) in air from the photo-oxidative stress caused by blue light (λ_max_ = 452 nm) taking the role of scavenger of reactive oxygen species (ROS) [15]. All in all, the photosensitization of ^1^O_2_ by irradiated betanin, and the antioxidant behavior of this same pigment, represent a paradox where dynamics must dominate the outcome.

In earlier studies, the strongly absorbing betanin has been described as a “non-fluorescent” pigment [16,17], which indicates the presence of efficient non-radiative conversion of light to heat; a convenient mechanism that proved useful in protecting plant leaves of the Caryophyllales order against UV light radiation [18]. This claim of being non-fluorescent was eventually adjusted and redefined as weakly fluorescent [19]. In fact, the reported paucity of photoluminescence arising from betanin drastically contrasts with the rich photophysical properties that it actually conveys. These traits include weak fluorescence and ultrafast S_1_ excited-state decays, fast electron and energy transfer processes, design of dye-sensitized solar cells (DSSCs) and organic light emitting diodes (OLEDs), nonlinear optical (NLO) materials, photocatalysis, photosensitization of ^1^O_2_ and its applications in PDT and antimicrobial activities, and photo-induced *cis-trans*-isomerization. This review describes the excited-state properties, both behavior and dynamics of betanin, which have been only sparingly reported over time. In fact, the literature shows that it is an emerging field, particularly in biotechnology [20]. In this review, three paradoxes are highlighted: (1) the ultrafast rate of internal conversion transforms over 99% of the absorbed light energy into heat, and yet, a short-lived fluorescence, photo-induced electron transfer and designs of DSSCs were reported; (2) the absence of intersystem crossing was noted, and yet photodynamic therapy was performed; and (3) while betanin is inclined at photosynthesizing ^1^O_2_, its intrinsic antioxidant behavior is also prone to trap this same species in an auto-sacrificial manner. Overall, betanin excited-state properties, which have demonstrated of being useful but underexploited, have been essentially ignored in past reviews.

***Fundamental photophysics***

The betanin natural dye has been known for quite a long time, first marked as a non-emissive and very photochemically sensitive species in water in the presence of oxygen. However, with time, this colorant turned out to exhibit far richer photophysical properties than anticipated. The following Section 2, Section 3, Section 4, Section 5 and Section 6 describe these properties and prepare the readers to better understand the forthcoming applications.

## 2. Optical Properties and Molecular Orbitals

**Figure 2:**
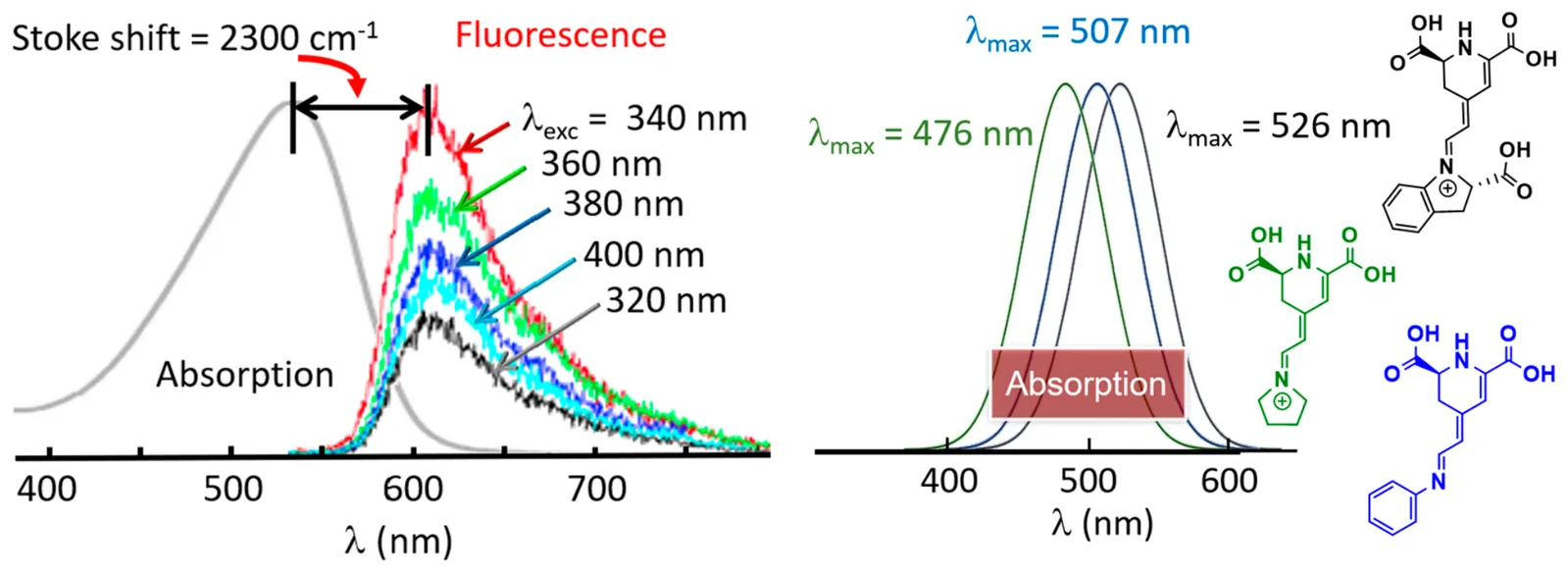
(**Left**) Absorption spectrum of betanin in water at 25 °C. Reproduced with permission from [21]. Fluorescence spectrum of betanin as beet root extract. Reproduced with permission from [26]. (**Right**) Representation of the lowest energy absorption band (Gaussian shape) of three selected betacyanins to stress the effect of the molecular structure on the position of the absorption maxima. Note that the structures were wrongfully drawn in the original manuscript. These structures have been corrected. The data are from [24]. Note that the shift in the 476 nm band (green) to 507 nm (blue) is due to the extension of the π-system upon adding the phenyl group, and to 526 nm (black) is due to the planarization of the π-system, thus optimizing the π-orbital overlaps.

**Figure 3:**
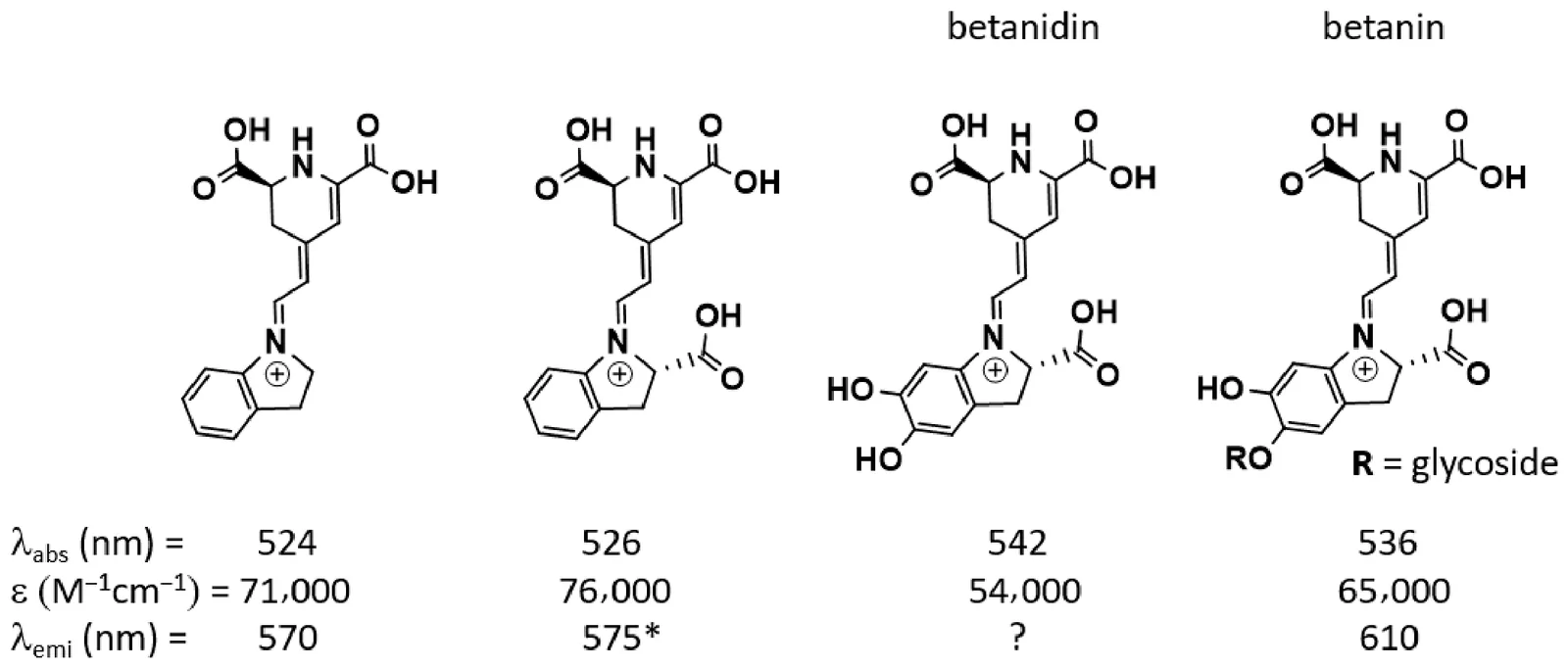
Selected optical parameters (absorption and fluorescence positions) and their absorption molar coefficient of selected betacyanins. Note that the structures were wrongfully drawn in the original manuscript. These structures have been corrected. The data are from [24]. * The relative emission intensity of the 2nd species (λ_emi_ = 575 nm) is ~20 times larger than that of the former (λ_emi_ = 570 nm). Note that upon anchoring electron-donating groups such as OH and OR on the benzene ring, the electronic density increases, thus destabilizing the HOMO manifold. By reducing the HOMO-LUMO gap, the absorption bands undergo bathochromic shifts. ? Also note that betanidin is not fluorescent according to [16]. An independent library search also turned out to be negative.

The absorption spectrum of betanin is characterized by an intense and broad band located at 535 nm (Figure 2) [21]. The absorption coefficient (ε) is 65,000 M^−1^cm^−1^ [22,23], meaning that the electronic transition is strongly allowed, which is consistent with its application as a food colorant. The origin of the reddish color and large ε value is due to the chromophore **phenyl-N^+^=C-C=C-C=C-CO_2_H**, which is relatively planarized by cycles within the molecular skeleton. This is well-illustrated in Figure 2 where the absorption spectra of three related betacyanins are compared, thus permitting to monitor the change in band position with the structural modification [24]. Based on the well-known Strickler and Berg relationship, which links a molecule’s ε value to its natural singlet emission lifetime [25], the large ε value of betanin suggests that the fluorescence lifetime will be most certainly short (i.e., <1 ns). Essentially, this relationship assumes the fact that in the absence of symmetry change or extensive distortion between the ground and excited state, the probability of absorption (S_0_ → S_1_) is the same as its fluorescence (S_0_ ← S_1_). Concurrently, upon substitution of various groups onto the bicyclic group, further bathochromic shifts in the absorption maxima are noted, all exhibiting large ε values (Figure 3) [24].

Betanin is weakly fluorescent and its first observation as a beet root extract was reported 17 years ago where λ_emi_ = 610 nm, using laser spectroscopy tuned with an optical parametric oscillator (OPO; Figure 2) [26]. The red-shifted position of the fluorescence is consistent with the series shown in Figure 3, and optimum emission intensity was obtained with λ_exc_ = 340 nm (Figure 2). The presence of fluorescence arising from betanin, also issued from beets’ extraction, was also subsequently confirmed (λ_emi_ = 608 nm) by another group [27]. The excitation spectrum exhibits a broad band with a maximum at 525 nm, which overall matches favorably the absorption spectrum. Figure 3 also illustrates two situations: (1) easily observable fluorescence for the first two examples, and (2) optically silent or very weak fluorescence for the last two (betadinin and betanin). The comparison indicates that the substitution on the phenyl group practically turns off the fluorescence. This phenomenon is known as the “loose bolt” effect, which is common when substitution is performed directly on benzene and aromatics in general [28]. The process is associated with an increase in the number of non-radiative deactivation pathways, such as vibrations.

**Figure 4:**
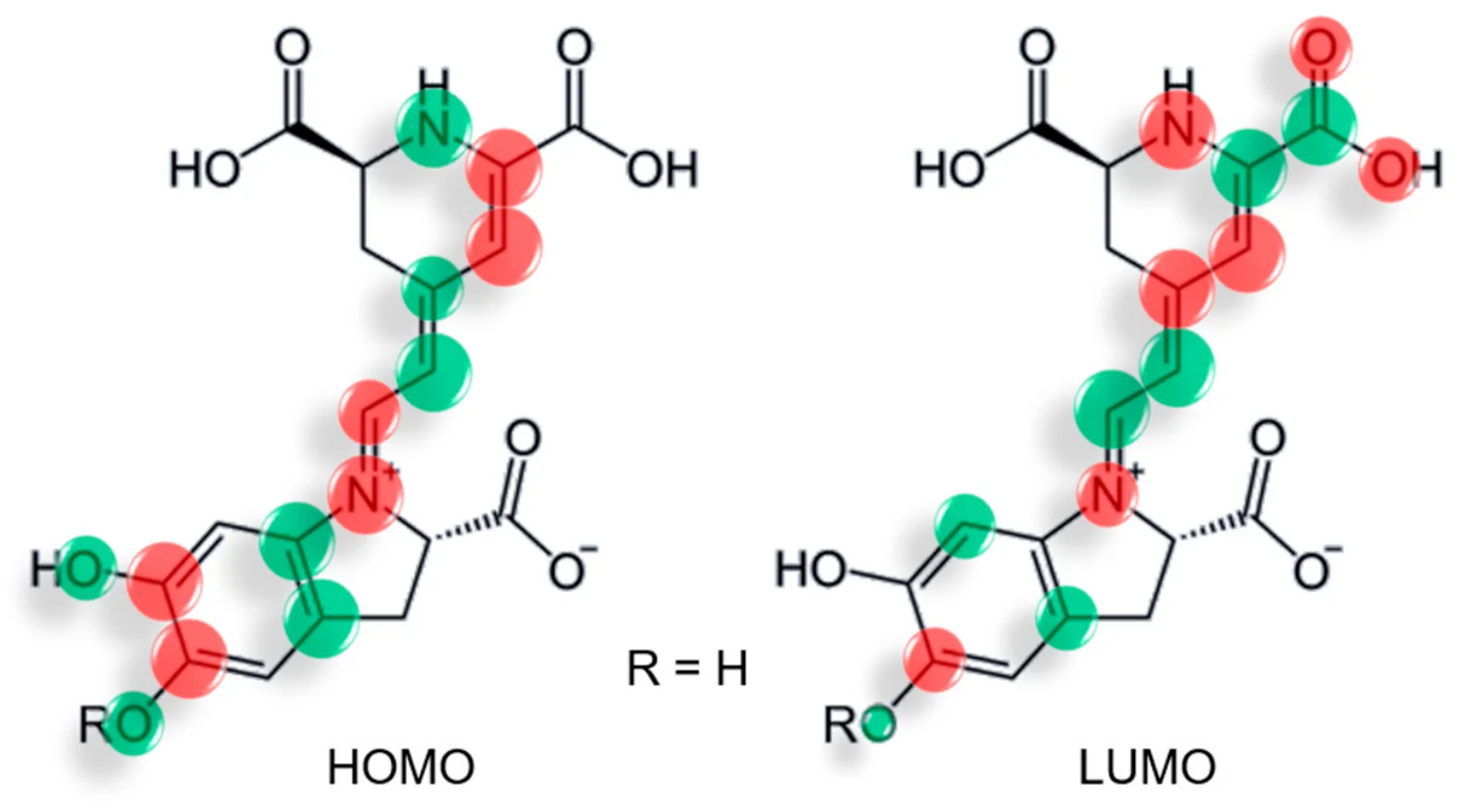
Representations of the frontier MOs of betanidin, which is assumed to be identical to that of betanin. Only the top phase of the p-atomic orbitals is represented.

The nature of the S_1_ excited state of betanin has been computationally addressed using density functional theory, DFT (Figure 4) [29,30]. The molecular orbital (MO) representations of the frontier MOs, HOMO and LUMO, indicate an atomic distribution over the phenyl-N^+^=C-C=C-C=C-CO_2_H chromophore through the π-system, which permits to safely assign the S_1_ state as a ππ* state via a HOMO→LUMO excitation. The calculated electronic density is evenly distributed from phenolic groups to the conjugated carboxylic acid. Moreover, the comparison of the atomic orbital contributions between the HOMO and LUMO indicates a larger proportion on the diphenolic portion in the HOMO, but a larger contribution on the conjugated C=C-CO_2_H end group. This situation leads to the formation to ππ* excited state with some charge-transfer character (CT) from the diphenolic group (push) to the C=C-CO_2_H withdrawing end group (pull) in the dihydropyridine moiety. This CT conclusion was recently further confirmed by subsequent DFT computations.

**Figure 5:**
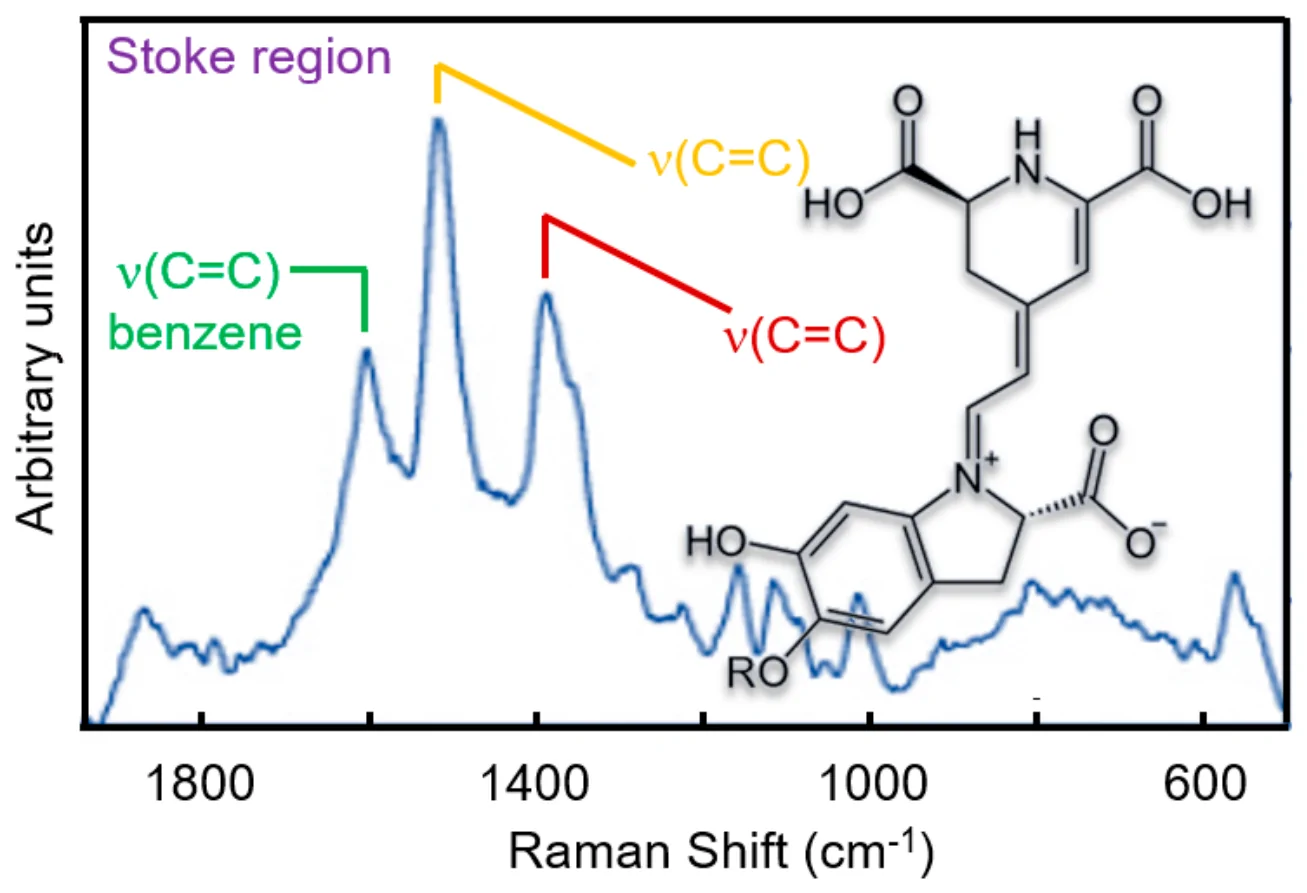
Resonance Raman spectrum of betanin (R = glucoside); λ_exc_ = 514.5 nm from an Ar(g) ion laser operating with a 10 mW power using a single monochromator and CCD detection. In this image, the background has been subtracted. The three main assigned peaks are 1387 (red), 1517 (yellow) and 1602 cm^−1^ (green).

The confirmation for this S_1_ assignment, being mainly ππ* singlet excited state, has been confirmed using resonance Raman spectroscopy (Figure 5) [27]. This technique consists of exciting the molecule using a wavelength matching the absorption spectrum and seeing which of the vibrational mode scattering is enhanced upon electronic resonance. The fact that betanin is weakly fluorescent turns out to be a positive property since fluorescence hinders the Raman signal. In this case, TiO_2_ nanoparticles were used to quench the residual fluorescence (by electron transfer, see below) thus conveniently optimizing the Raman signal. At the end, the three most intense Raman peaks are located at 1387, 1517, and 1602 cm^−1^, which are readily assigned to conjugated ν(C=C) modes and ν(C=C) of the benzene group.

## 3. Excited-State Dynamics and Quantum Yields

**Figure 6:**
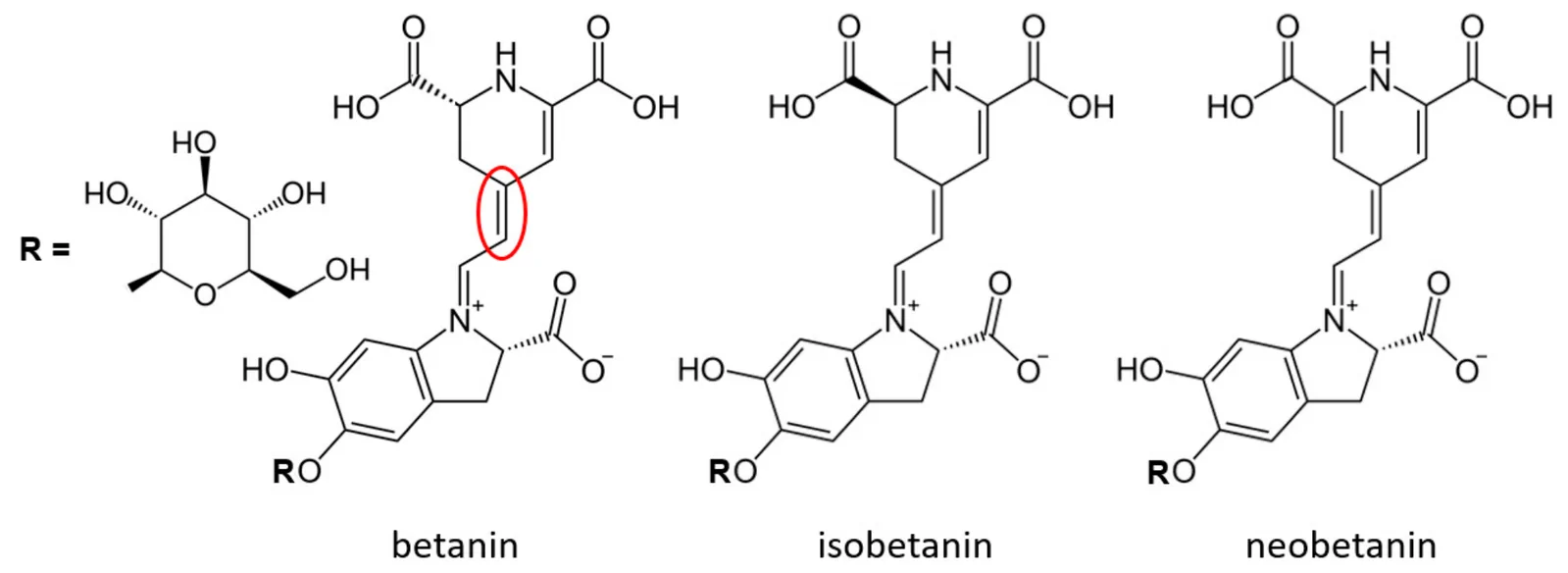
Comparison of the molecular structures of betanin, neobetanin and isobetanin. The encircled double in red is where the *cis-trans*-photoisomerization takes place based on the information provided in [34].

The first measurement of the fluorescence lifetime (τ_F_) and quantum yield (Φ_F_) were reported just over a decade ago [31,32]: Betanin exhibits of τ_F_ of (τ_S1_ from fs-transient absorption spectroscopy with the stimulated emission signal) 6.4, 7.7, and 27 ps, and Φ_F_ data of ~0.0007, 0.0013, and 0.0046 in water (0.89), methanol (0.54) and ethylene glycol (viscosity of 16.1 cP), respectively. Consequently, the corresponding radiative rate constants (k_F_ = Φ_F_/τ_F_) are ~1.1 × 10^8^, 1.7 × 10^8^, and 1.7 × 10^8^ s^−1^, respectively, for the three solvents. These values compare favorably with that of 1.8 × 10^8^ s^−1^, which was calculated with the Strickler and Berg equation [25]. Conversely, the non-radiative rate constants (k_nr_ = (1 − Φ_F_)/τ_F_) are ~1.6 × 10^11^, 1.3 × 10^11^, and 0.37 × 10^11^ s^−1^, for water, methanol, and ethylene glycol, respectively. These values are in the same order of magnitude to that reported for neobetanin (k_nr_ ~ 4.3 × 10^11^ s^−1^ in H_2_O) [33]. These viscosity-based experiments provide a solid argument to state that the S_1_ excited state is very efficiently deactivated by molecular motions and that betanin converts light energy into heat very efficiently (>99%), providing this dye the interesting property of plant protector against light-induced stress. This hypothesis has been confirmed by comparing the photophysical properties (λ_F_, τ_F_, Φ_F_, etc) of betanin, neobetanin (14,15-dehydro-betanin) and isobetanin (Figure 6) in water, d_2_-water, methanol, and ethylene glycol, as these molecules share common structural characteristics [33]. These three molecules exhibit very similar viscosity-dependent photophysical properties. Parallel studies on betalains using water/trifluoroethanol mixtures as media also reported the same Φ_F_ data changes with viscosity increment [34].

**Figure 7:**
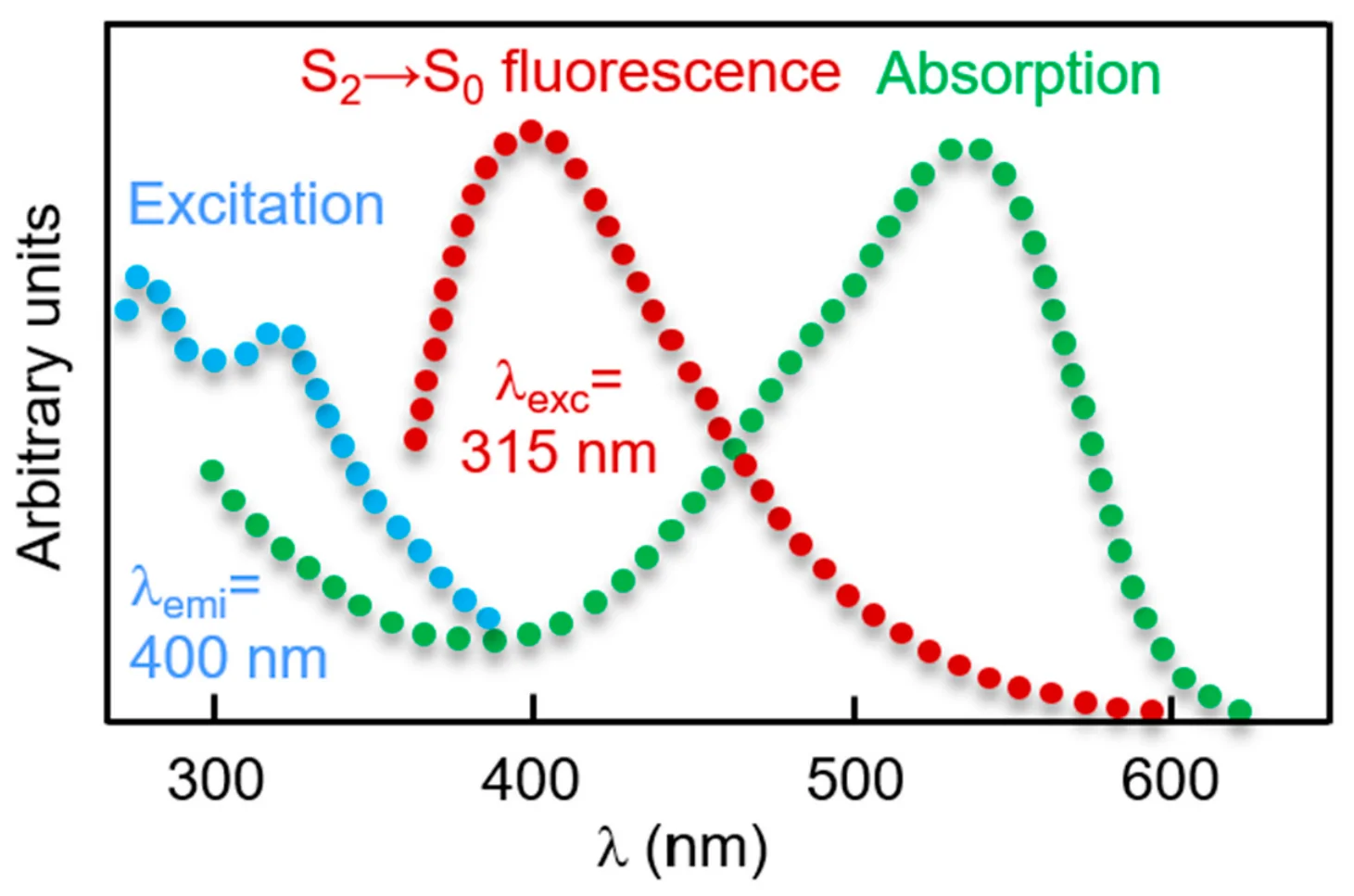
Representation of the absorption (green), S_2_ → S_0_ fluorescence (red wine); λ_exc_ = 315 nm (UV-A), and excitation (turquoise) spectra of betanin in water in the presence of a little bit of acetic acid. The λ_abs_ of betanin is 535 nm. The original data to generate this figure are from [18].

In addition, a Soret (or anti-Kasha) fluorescence (S_2_ → S_0_) has also been reported for protonated betanin in the presence of acetic acid (blue line in Figure 7), while betanin in water alone is non-fluorescent (black line) [18]. The fluorescence decay is biphasic: major component with τ_F_ = 29 ps (relative intensity of 91%), minor component with τ_F_ = 9 ps (relative intensity of 9%). The magnitude of the lifetime values corroborates the low emission intensity, which is reminiscent of that of the S_1_ state. Anti-Kasha fluorescence emissions are a rare phenomenon, and it is often associated with a very large energy gap between the S_2_ and S_1_ state, like the classic example of azulene [28]. The large energy gap between these two states in Betanin is well-illustrated in Figure 7 where the 535 nm band (S_0_ → S_1_) is definitely placed far from that of the S_2_ (i.e., S_0_ → S_2_), which is located at λ > 300 nm. However, the nature of the data may have to be taken with a little grain of salt since betanin is sensitive to acids as betanin undergo a slow dehydrogenation (to form neobe-anin) or a deglycosilation (to form betanidin) reaction [35]. Nonetheless, the presence of this radiative process, S_2_ → S_0_ emission, provides a tool to betanin acting as a phytoprotective molecule as it diverts light energy away from photo-destructive processes damaging the plants. Interestingly, the use of 2′-O-apiosyl-6′-O-crotonic acid–betanin inside an OLED showed the presence of a broad emission located at ~392 nm, reminiscent of that illustrated in Figure 7 [36]. Protection against UV-induced damage in HaCaT cells for skin cancer chemo-prevention has also been demonstrated [37].

No phosphorescence has ever been detected for betanin. This outcome is consistent with transient absorption spectroscopy that indicated that no intercrossing system occurs in this dye [32]. This conclusion was also obtained subsequently by another group [38].

## 4. *cis-trans*-Photoisomerization

The very efficient non-radiative deactivation process described above stems from fast rate of internal conversion, k_ic_ (collisional, vibrational), accompanied by ultrafast *cis-trans*-photo-isomerization estimated to be responsible for 50% of the depletion of the S_1_ population [33]. This investigation stressed the fact that the chromophore had to go through an intermediate twisted state, then followed by a fast molecular relaxation through a conical intersection, to explain the photophysical data. It is noteworthy that *cis-trans* isomerization can also occur via thermal and sonication paths [39]. At room temperature, betanin exists into two forms: *E* (*trans*) and *Z* (*cis*) in an 80:20 ratio, most probably through a combination of thermo- and photo-stationary states [40]. Concurrently, the isomerization of betanin into isobetanin is also known to occur upon steaming samples [41]. However, this process has never been observed to be photochemically induced.

## 5. Singlet–Singlet Energy Transfer

**Figure 8:**
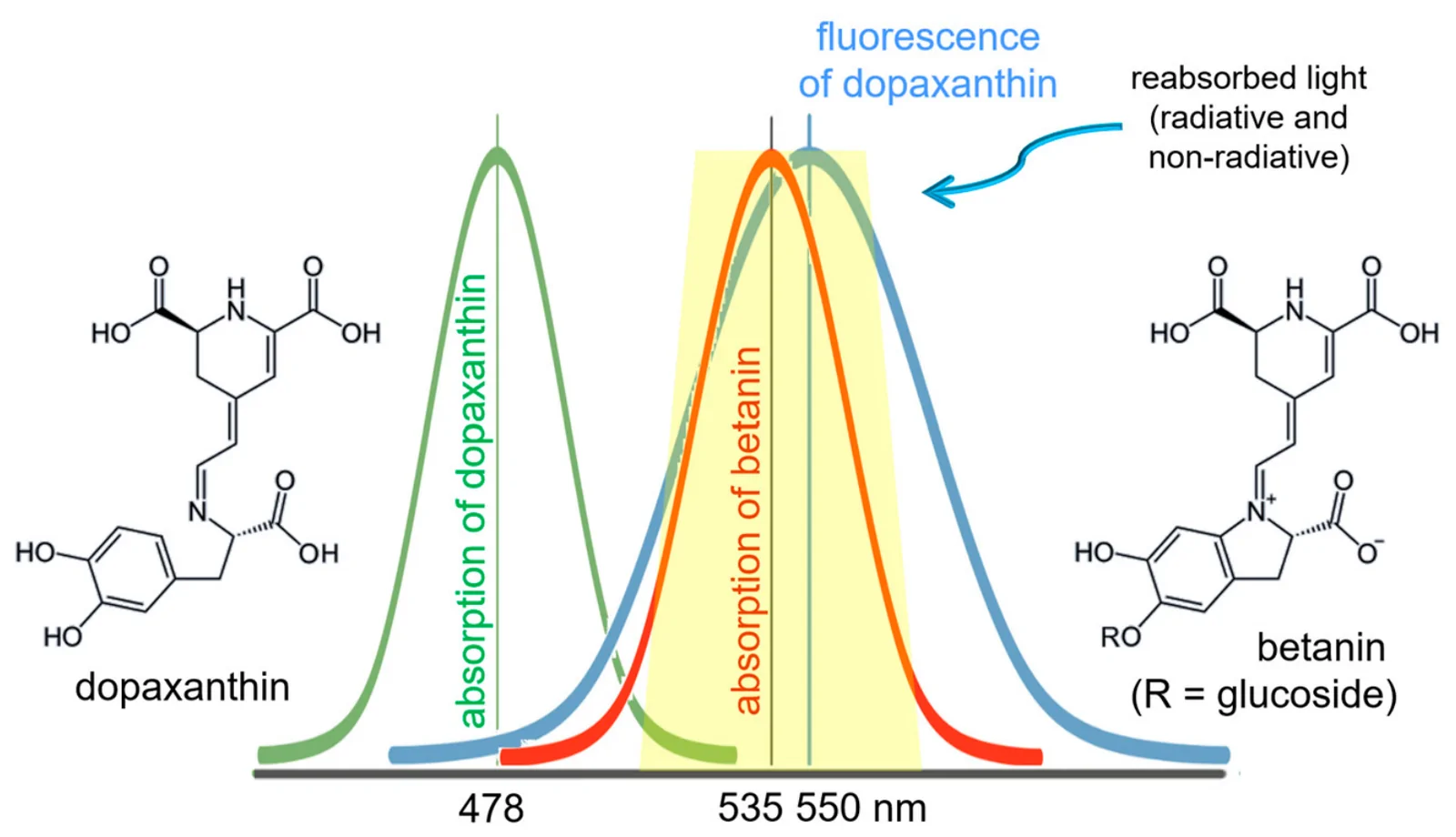
Schematic comparison of the absorption spectra of dopaxanthin (green line) and betanin (red line) and fluorescence spectrum of dopaxanthin (blue line). The yellow region is the range the emitted photons are reabsorbed. The maxima values placed at the bottom of the graph are from reference [24].

Many flowers contain yellow betaxanthins (463 < λ_abs_ < 474 nm), which are also fluorescent (509 < λ_abs_ < 512 nm), thus rendering the flowers emissive, thus providing specific emitted wavelengths to attract pollinators [6]. The presence of violet betacyanin, namely betanin, in the vicinity of betaxanthins, plays the role of a spectral modulator “creating a contrasting fluorescent pattern on the flower’s petals” [5]. An example is provided in Figure 8. The modulation of the light emitted by the petals can occur via two mechanisms: radiative and non-radiative.

❶ The radiative pathway consists of an absorption of light emitted by the xanthin lumophore by the betanin chromophore. The fluorescence wavelengths (green line; Figure 8) match perfectly with those of the absorption spectrum of betanin (purple line). However, the yield of this process is not 100% as the directions of the emitted light do not necessarily include a betanin absorber being physically placed in every path. Moreover, there is also a highly probable mismatch of the orientation of the transition moments of the light emitter (xanthin) and absorber (betanin). Only parallel orientations of the transition moments lead to an absorption of the emitted light. All in all, this geometric situation leads to a less efficient process.

**Figure 9:**
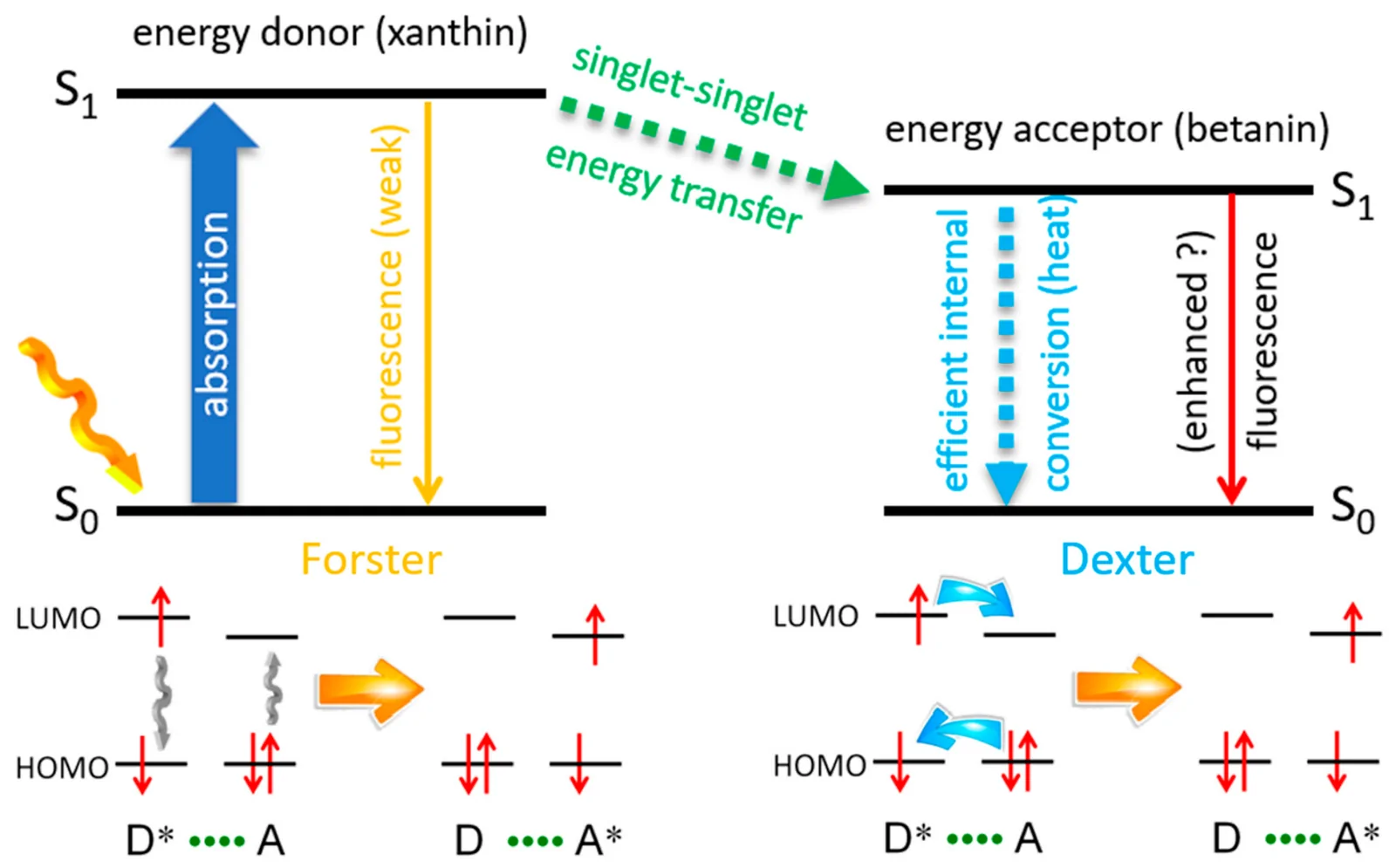
(**Top**): State diagram showing the process of singlet–singlet energy transfer, applied to a betaxanthin (xanthin)-betanin dyad. (**Bottom**): Illustration of the Forster and Dexter mechanisms.

❷ The non-radiative path consists of an energy transfer from the excited donor (betaxanthin) in its first excited state to the relaxed acceptor (betanin, S_0_, Figure 9 top) that may occur in two possible mechanisms: Forster (fluorescence resonance energy transfer, FRET) or Dexter (Figure 9 bottom) [42,43]. As the S_1_ state of betaxanthin is depleted, that of betanin is increased. The expected increase in the betanin fluorescence was not verified [5]. Nonetheless, the known efficient conversion of light energy into heat by betanin renders the latter an efficient protecter of the plants from oxidative stress and caused excess of excited chromophores within its membranes.

The process of phytoprotection by betanin contained in djulis, for instance on retina cell (ARPE-19 cells), is well-established, but the complete biological mechanism inside the cells far exceeds this primary singlet–singlet energy transfer process [15]. Nonetheless, this step is the first one to occur and triggers subsequent biological activity.

The Forster process stems from interactions between a donor and acceptor. Their strength depends on the magnitude of their transition dipole. This theory states that the rate of FRET, k_ET_, is given by Equation (1):

k_ET_ = k_F_^0^(D) • (κ^2^/r^6^) • cte • J (1)

where the terms k_F_^0^(D) (radiative rate constant of the energy donor in the absence of energy transfer), cte (a constant), and **J** (a normalized overlap integral) are given by Equations (2)–(4). The r value is the center-to-center distance between the donor and the acceptor.

k_F_^0^(D) = (Φ_F_^0^(D)/τ_F_^0^(D)) (2)

cte = (9000 • ln10)/(128 • π^5^ • n^4^ • N_a_)) (3)

**J** = (Φ_Δ_(λ) • ε_A_(λ) • λ^4^ dλ)/(Φ_Δ_(λ)dλ) (4)

where Φ_F_^0^ and τ_F_^0^ are the fluorescence quantum yield and lifetime, respectively, of the donor in the absence of energy transfer, N_a_ is the Avogadro’s number, n is the refractive index of the medium, F_D_ is the fluorescence intensity at the given wavelength (λ), and ε is the molar absorption coefficient at the same λ (nm).

Finally, and *κ*^2^ is an orientation factor between the directions of the transition moments of the donor and acceptor and is given by Equation (5):

*κ*^2^ = (sin*θ_D_* sin*θ_A_* cos*φ* − 2cos*θ_D_* cos*θ_A_*)^2^ (5)

where *θ_D_* is the angle made by the transition moment vector of the donor and the center-to-center vector, *θ_A_* is the angle made by the transition moment vector of the acceptor and the center-to-center vector, and *φ* is the dihedral angle between them.

Concurrently, the Dexter mechanism stems from a double electron exchange between the excited energy donor and relaxed acceptor. This theory states that the transfer rate, k_ET_, is given by Equation (6):

k_ET_ = K J exp(−2r/L) (6)

where K is an arbitrary constant and L is the average van der Waals radii of the donor.

The dominant mechanism is essentially defined by L. If r < L, then Dexter dominates. If r > L, then FRET dominates [44]. The three most significant parameters are r (due to its sixth power), *κ*^2^ which may vary between 0 and 4, and in a lesser extent, **J**, which was well-illustrated in the literature [45]. Again, this **J**-integral for betanin is well-illustrated in Figure 8. These three parameters provide easily controllable tools to adapt to the photo-oxidative stress endured by plants. Upon stress, plants can bio-synthesize more or less photo-protective molecules such as betanin and perhaps control also the relative orientations. Because dyads in nature tend to be separated from each other by more than the L value, the FRET process is the most encountered and can extend over 30 nm. Conversely, the opposite occurs in the solid state where the photo-active site are mostly in contact with each other [43].

**Figure 10:**
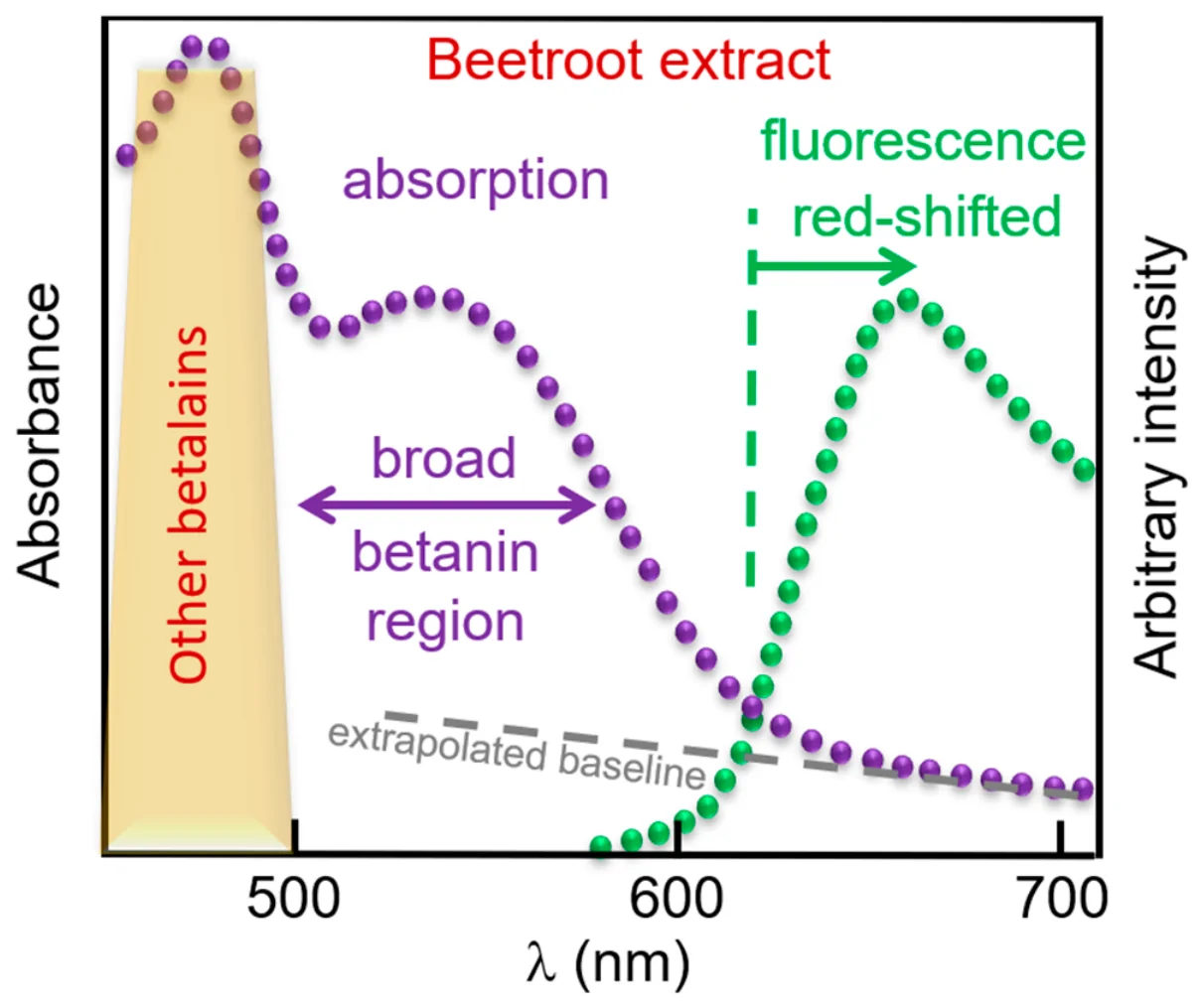
Representation of the absorption (purple) and fluorescence (green) spectra of red beetroot extract in EtOH. The data used to reconstruct the spectra are from [46]. For this mixture, the Commission Internationale d’Eclairage, CIE, coordinates are {0.65, 0.34}.

Clear evidence for the presence of singlet-to-singlet energy transfer (FRET) in solution containing betanin was provided directly with red beetroot extract [46]. This cocktail contains several betalain dyes, including betanin, characterized by different absorption (and fluorescence) maxima. Figure 10 exhibits both absorption and fluorescence spectra of beetroot in EtOH (10^−5^ M). It is noteworthy that the higher viscosity of EtOH compared to that of water will promote higher τ_F_ and Φ_F_ values for betanin (see Section 3). By comparison with Figure 2, the absorption zone between 450 and 500 nm exhibits a strong feature at ~475 nm indicating the presence other betalain chromophores in solution. The emission trace, which starts at 525 nm, shows no emission until 600 nm. Then, an emission peaking at 660 nm, reminiscent of that of betanin (λ_emi_ = 610 nm; Figure 2) is depicted. It is noteworthy that spectral anomalies indicate the presence of aggregates: (1) both absorption (~10 nm) and fluorescence (~50 nm) spectra are red-shifted with respect to betanin shown in Figure 2 (left), and the Stoke shift is 3200 cm^−1^ (instead of 2300 cm^−1^); (2) the baseline is not flat, so particles are formed. These observations indicate that the concentration used is too large (10^−5^ M). The broadness of the betanin band is due to the overlap with betalains’ bands.

The absence of high energy fluorescence, and a stronger signal associated with betanin is a clear signature of a singlet–singlet energy transfer. In this investigation, the authors did not notice these features. However, by mixing java plum and beetroot (Commission Internationale d’Eclairage, CIE, coordinates {0.32, 0.34}), and mixing carrot and beetroot {(0.33, 0.33}) in solution and in gel medium (the CIE values are {0.31, 0.34} and {0.33, 0.32}, respectively), white light emissions were obtained. In the mixtures in gel, the donor*-to-acceptor processes are (anthocyanin* to betanin) and (β-carotene* to betanin), respectively. With the knowledge of their center-to-center D**^…^**A separations, their k_ET_ values have been estimated: 2.78 × 10^9^ s^−1^ (r = 0.5 nm) for the former and 1.02 × 10^8^ s^−1^ (r = 0.4 nm) for the latter. These orders of magnitude compare favorably to what is reported for loosely connected dyads in the literature [47,48].

## 6. Photo-Induced Electron Transfer

After several singlet–singlet energy transfer hops between chlorophylls, the photo-induced electron transfer between the special pair and the pheophytin is the primary process occurring within the photosynthetic membranes of plants. In a more general sense, it can occur in two manners; either the excited donor transfers its electron (D* + A → D^+^^•^ + A**^−^**^•^) or the excited acceptor abstract an electron (D + A* → D^+^^•^ + A**^−^**^•^). They are called photo-oxidative and photo-reductive mechanisms, respectively. Beside the photosynthetic membrane, organic solar cells (OSCs) also depend on this process. For OSCs containing a non-fullerene electron acceptor (NFA) and a conjugated polymer donor, for instance, in the active layer of the bulk heterojunction solar cell, both mechanisms occur simultaneously, thus desirably optimizing power conversion efficiency (η).

The inexpensive betanin electron-donating pigment has also been used as a photo-active component in devices. For example, the nano-structured thin film of the semi-conductor hematite (α-Fe_2_O_3_) with dip-coated betanin onto a fluorine-doped tin electrode (FTO) substrate as a photoanode, was performed [49]. The attachment of betanin to hematite occurs through coordination bonding between one or more of the carboxylate groups and the Fe(III) centers. This supposition is corroborated from DFT computations where the proton affinities of the three carboxylic groups are computed [50]. The one with the lowest affinity is the (only) conjugated one -CH=CCO_2_H, for which the stabilization of the conjugated base occurs through charge delocalization.

**Figure 11:**
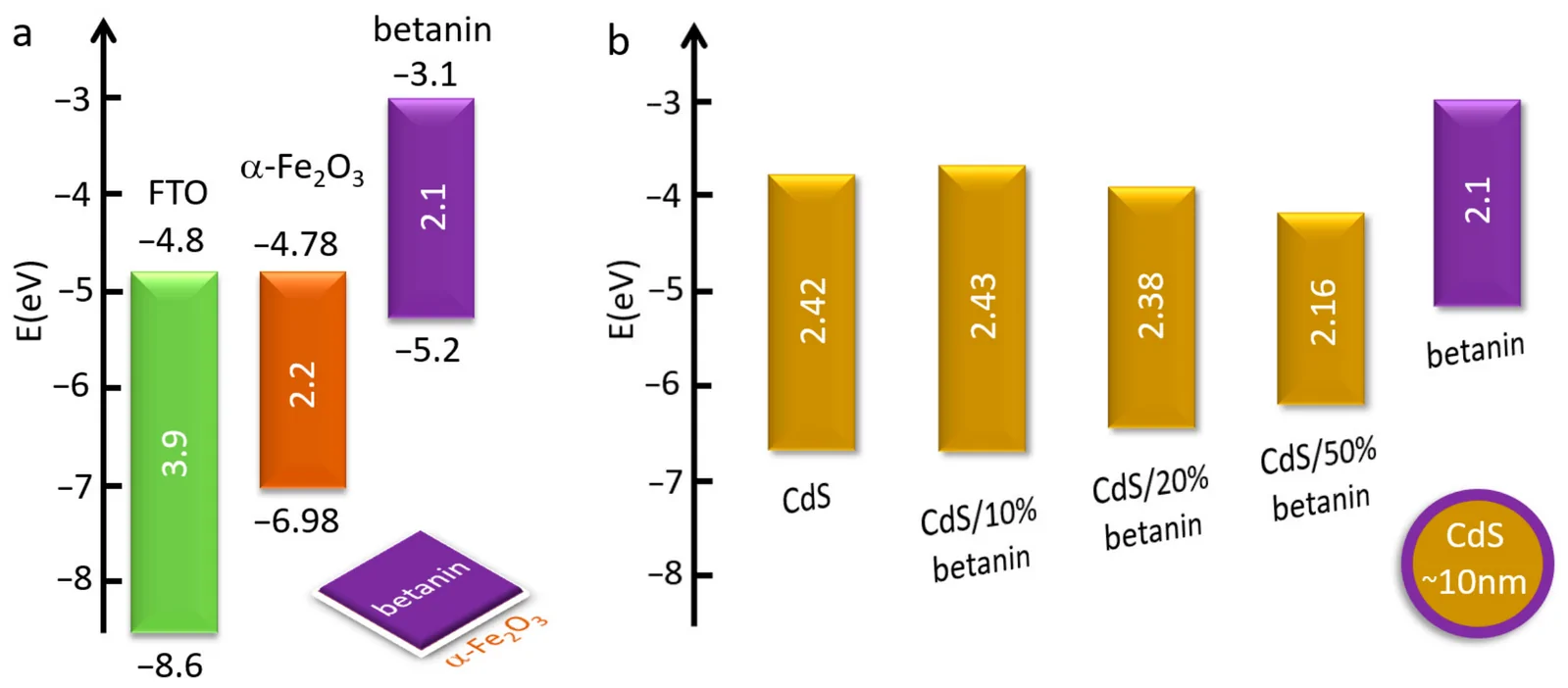
Comparison of the band gaps (between the valence band (VB) and conduction band (CB) of a nano-structured thin film of hematite dip-coated with betanin on an FTO glass [49] (**a**) and of the nano-sized composites of CdS particles coated with betanin (**b**). The intermediate bands are 1.89, 1.88, and 1.62 eV for 10, 20, and 50% loading, respectively [54].

The possible photo-oxidation reaction in water stems from the intrinsic antioxidant properties. The energy band diagram is provided in Figure 11a. The hematite photoanode alone cannot photochemically split water. The primary step consists of betanin* + hematite → betanin^+^^•^ + hematite**^−^**^•^, and the photocurrent density augmented from 0.0798 mA/cm^2^ at 0.5 V for pristine hematite to 0.19 mA/cm^2^ (after 24 h after usage). Note that the scale is for vacuum and that the level for I_3_^−^/I^−^ electrochemical couple is near −5.0 eV rendering betanin an appropriate ingredient for dye-sensitized solar cells (DSSCs).

This process is accompanied by a decrease in the photoluminescence lifetime of the pigment from pristine (in the solid state; 2 ns) to coated form (0.11 ns), indicating the presence excited-state quenching, which is consistent with an electron transfer process. Interestingly, τ_F_ is 2 ns in the solid state, whereas in solution, it is at best 0.027 ns in ethylene glycol (see above). This two-order of magnitude change is significant and consistent with the effect of viscosity, but also the constraint imposed by the solid state prevents photo-induced *cis-trans*-isomerization, thus removing an important channel of excited-state deactivation. The rate of electron transfer, k_et_, can also be evaluated from k_et_ = (1/τ_F_) − (1/τ_F_º) where τ_F_ and τ_F_º are the fluorescence lifetimes, respectively, in the presence and absence of electron transfer [51]. In this case, k_et_ is found to be 8.6 × 10^9^ s^−1^, which is in the typical range for electron transfer in general, but is on the slow side for solid state processes such as at the interface of an electron donor and acceptor in an OSC (fs/ps-timescale) [52,53].

Similarly, the dip-coated bio-functionalization of cadmium sulfide nanocrystals of 8 to 17 nm dimension (from Transmission Electron Microscopy measurements) was performed [54]. This functionalization permits an impressive gain in both dark and photoconductivity compared to that of CdS alone (the energy band diagram is provided in Figure 11b). The binding of the organic pigment to the nanoparticles (NPs) seems to occur through the S-atom in the composite based on FTIR spectroscopy but remains unsure.

**Figure 12:**
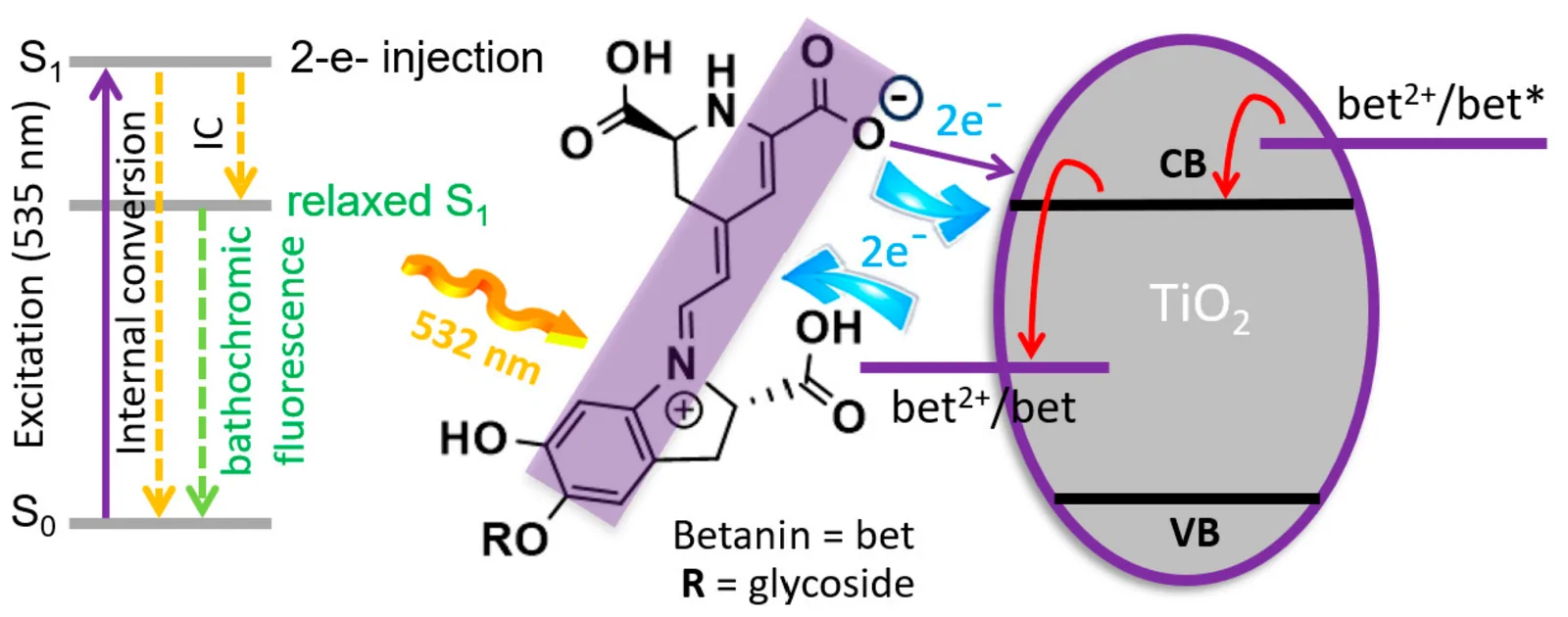
(**Left**) Cartoon illustrating the state diagram and photophysical events in the betanin/TiO_2_ NPs system. (**Right**) A proposed 2-electron mechanism for the photo-oxidation of betanin on TiO_2_.

The timescale of photo-induced electron transfer between TiO_2_ NPs (anatase) and ZrO_2_ NPs (particle sizes of ~20 nm) dip-coated with betanin forming together a thin film, was assessed by fs-transient absorption spectroscopy, fs-TAS (Ti-sapphire; FWHM = 50 fs; λ_exc_ = 580 nm) [55]. The primary feature is that all transient species are already formed within the laser pulse. Then, the electron transfers (called electron injections) from betanin to TiO_2_ and ZrO_2_ NPs occur, thus producing supplementary relaxation in the transient decay. The decays are biphasic, exhibiting a fast (~1 ps) and slow component (~6–8 ps), which can be associated by a unimolecular relaxation of betanin, but also involving a depopulation of the S_1_ state by electron transfer to the NPs (Figure 12). The ~6–8 ps component is reminiscent of the τ_F_ value for betanin discussed above (Section 3) [31,32]. The ~1 ps value for the depopulation of S_1_ betanin* associated with electron transfer permits to evaluate k_et_ ((1/τ_F_) − (1/τ_F_º)), which is ~8.6 × 10^11^ s^−1^. This value is in line with what is commonly encountered for solid state samples [52,53]. The “complex” interpretation of the combined fs- and ns-TAS, and DFT computation data for the betanin/TiO_2_ NPs case consists of a simultaneous double electron injection from betanin* to TiO_2_ NPs (Figure 12) and is accompanied by the formation of an oxidized product, 2-decarboxy-2,3-dehydrobetanin. This outcome contrasts with the betanin/ZrO_2_ NPs case for which no photoproduct is observed, and a classic one-electron injection occurs.

**Figure 13:**
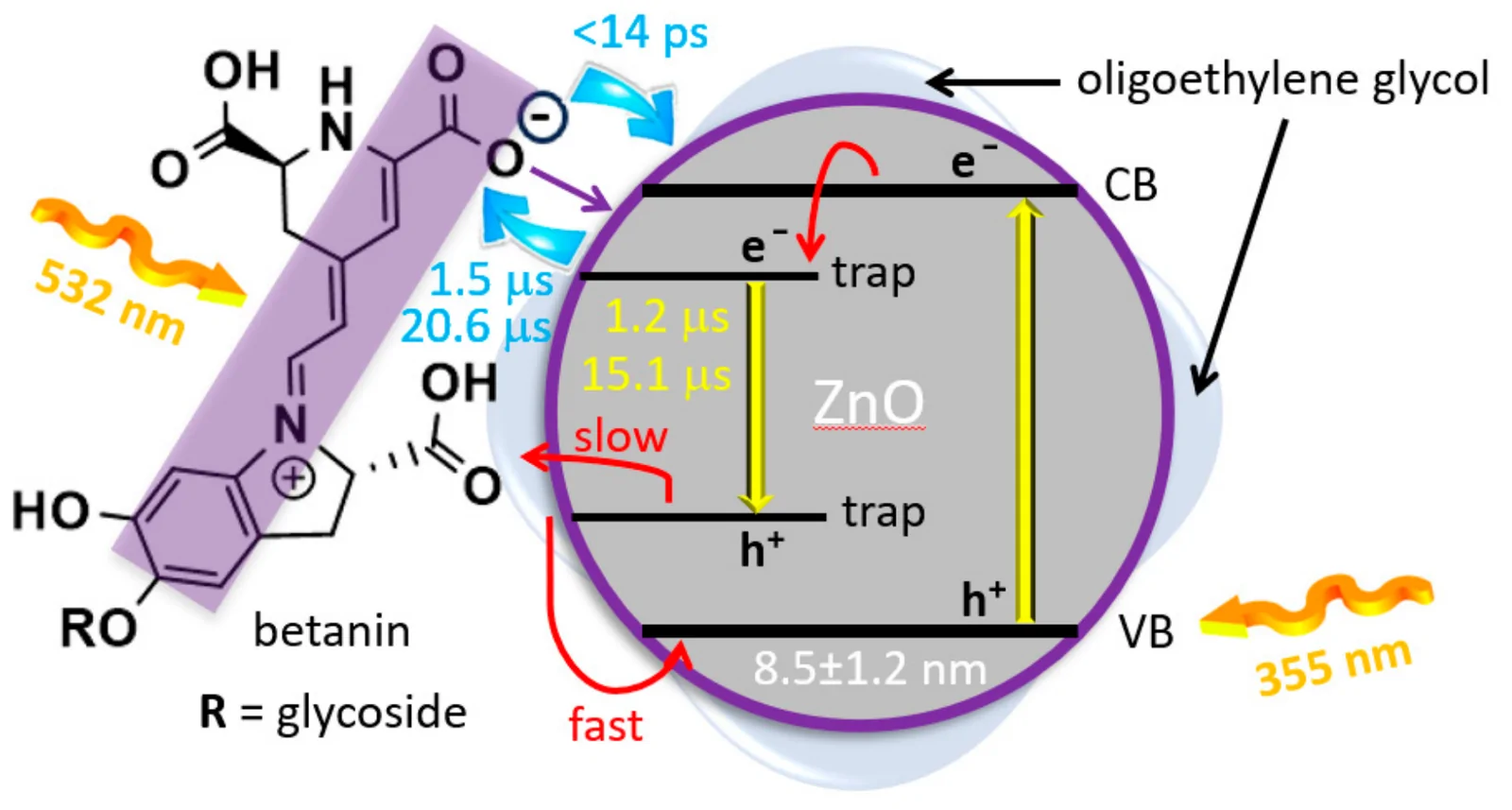
Scheme representing the comparison of the timescales of the photo-induced electron injection and recombination in betanin-ZnO NP (stabilized by carboxylate-EOG) [56]. The techniques used are a combination of time-resolved fluorescence, fs-transient UV-vis and ns-transient infrared spectroscopy.

In order to exploit the photo-induced electron transfer process for applications, for example, in solar cells, the charge-separated state must be long-lived to minimize the probability of charge recombination at the dye/NP interface (dye^+^^•^/NP**^−^**^•^ → dye/NP) and simultaneously increase the probability of charge transport across the photoanode. This timescale was evaluated for ZnO NPs (~8.5 nm) stabilized by carboxylate ethylene glycol (carboxylate-EOG; for aqueous solution purposes) and combined with betanin, a photosensitizer [56]. Using a combination of time-resolved fluorescence, fs-transient UV-vis and ns-transient infrared spectroscopy, the complete photophysical behavior of the NP composites was elucidated (Figure 13). Upon exciting in the maximum of the betanin absorption band at 532 nm, the photo-injection of an electron into the Zn NPs occurred within 14 ps, which corroborates that reported for TiO_2_ and ZrO_2_ NPs (~1 ps). Then, recombination occurs in a biphasic manner in the μs timescale (84% at 1.5 μs and 16% at 20.6 μs). Concurrently, the direct excitation of the ZnO NP containing no betanin (just carboxylate-EOG) at 355 nm leads to the excitation of the ZnO NP and its relaxation is also bi-exponential (91% at 1.2 μs and 9% at 15.1 μs). The magnitude of these times and relative amplitudes indicates that the ZnO NP characteristics play a major role in the outcome. In this case, the μs prior to recombination in the betanin-ZnO NP system is particularly long-lived compared to what is expected; ns-timescale was deduced from soluble models and fs-transient absorption spectroscopy [57,58,59]. All in all, the slow to very slow recombination processes permit charge and hole transports to occur, which open the door to solar cell applications.

***Applications***

Section 7, Section 8, Section 9, Section 10, Section 11, Section 12 and Section 13 present the various betanin-based applications. These investigations have been mostly reported over the past decade or so. Despite the kinetically “tight conditions” of the excited-state properties of betanin as illustrated above, an interesting list of possible applications have been reported.

## 7. Dye-Sensitized Solar Cells

The photo-oxidative process of betanin easily and rapidly undergoing under light irradiation provides the opportunity to fabricate solar cells, notably DSSCs. Considering that this dye is abundant, inexpensive, environmentally friendly and non-toxic, this endeavor is attractive despite its intrinsically challenging ultrafast internal conversion rate, k_ic_. However, this process competes with the also ultrafast photo-induced electron injection into the semi-conductor, and processes occur precisely within the same timescale, as discussed (ps) [55]. This phenomenon leading to the undesired consequent lower η values was precisely discussed when betalains (extract from beetroot: betanin (major), 2,17-bidecarboxybetanin, vulgaxanthin I, and betanidin-6-O-(6′,6″-di-O-trans-4-coumaroyl)-β-sophoroside) were used as a cocktail to fabricate DSSCs [60], and the resulting η values are modest and far from what would be considered near top values of ~15% for modern DSSC technology [61]. This outcome is stubbornly constant despite the cocktail employed containing various natural dyes [62]. It easily demonstrated that the engineering manner in which the cells are constructed plays a significant role in the resulting η [63] and the nature of the betanin-containing cocktail used as dye source (1.36 < η < 3.48%; in this study the natural dyes used in various cells were poorly identified) [62]. Nevertheless, DSSC devices were also fabricated using betanin as the main or only dye, and a list of investigations is provided in Table 1, stressing on the components used, (best) η obtained, and year of report [21,27,45,50,64,65,66,67,68,69,70,71,72,73,74,75,76,77,78,79,80,81,82,83,84,85,86,87]. The largest h’s (h > 3) were obtained over the past 5 years [62,64,67,76,83]. The use of cocktails of dyes has the great advantage of exhibiting a very large absorption window of visible light, thus optimizing light harvesting. Parallel studies consisting of doping the betanin-containing TiO_2_ NPs with various metals such as Al [72] and Mg [88] have also been performed. Finally, exceptionally high η values ranging from 5.82 to 14.21% were also reported where the TiO_2_ NPs (20 to 50 nm) exhibited 1D nanorods assembling together to give a 3D nanoflower-like architecture, but looking at the photocurrent–voltage curves, one may want to take this report with a grain of salt [89].

## 8. Organic Light Emitting Diodes

In contrast to solar cells, OLEDs are devices in which electric energy from an applied voltage is fed into the devices, which in return produce light. The first electrochemical reaction consists of generating charges and holes at the cathode and anode, which then migrate across multilayered device, and when they meet at an interface, the subsequent recombination reaction takes place: D^+^^•^ + A**^−^**^•^ → D + D* + A, where D is the easily oxidized species, the betanin chromophore. Betanin is also known to be a semi-conductor in the solid state, which is an essential criterion [90]. The appearance of D in the ground state (S_0_) is common, even more for betanin due to its efficient intrinsic non-radiative processes. D* is the same chromophore but has acquired some electric energy to populate excited states (S_n_, and T_n_, n > 0). Based on the electron spin recombination, 25% of the populated excited states of D* should lead to singlet (↑↓) and 75% triplet states (↑↑, ↓↓, ↑↓).

**Figure 14:**
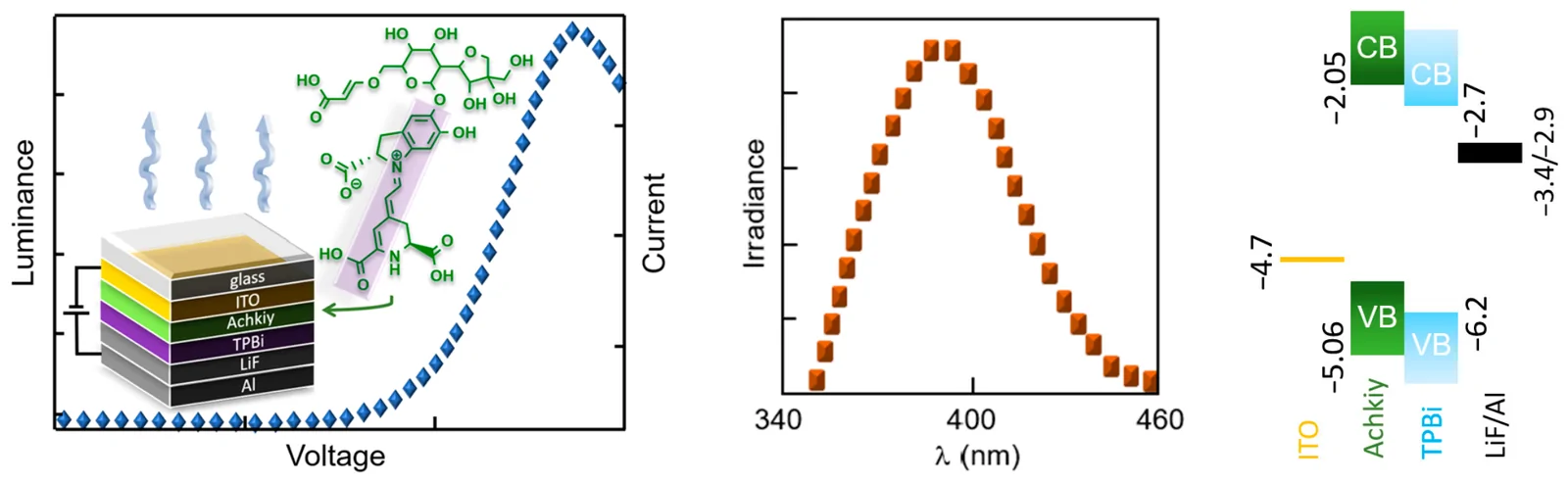
(**Left**) General representation of the luminance vs. voltage of the OLED devices containing **Achkiy** and the OLED architecture; note that the reported molecular structure of **Achkiy** is uncertain due to the unspecified *E* vs. *Z* isomerism. Note the striking resemblance of the **phenyl-N^+^=C-C=C-C=C-CO_2_H** chromophore in **Achkiy** (shown with a purple window) with that of betanin. The separations of the voltage, current, and luminance are, respectively, 6 V, 10 mA, 1 Cd/m^2^. (**Center**) Qualitative representation of the electroluminescence of the device (the separation corresponds to 20 μW/m^2^). (**Right**) Illustration of the band gap energy diagram deduced from calculations. The curves have been reconstructed from data of [14].

A recent example is the used of 2′-O-apiosyl-6′-O-crotonic acid–betanin (Achkiy) isolated from the ayrampo seed cuticle [36]. Its structure consists of a betanin, except that the R group is a 2′-O-apiosyl-6′-O-crotonic residue (Figure 14). In brief, its photophysical properties are still not reported so far, but the absorption spectra exhibit a maximum at λ_abs_ = 534 nm, which is expectedly identical to that of betanin. Conversely, this molecule turns out to be photoluminescent in the visible region as a thin film. The presence of glucose residues induces H-bond-driven aggregations in the solid state, which is perceptible by AFM, which also indicates a roughness of ~10.84 nm.

The OLED architecture is glass/ITO/**Achkiy**/TPBi/LiF/aluminum is shown in Figure 14 ((TPBi = 2,2′,2′′-(1,3,5-benzinetriyl)tris(1-phenyl-1-H-benzimidazole)). Based on calculations, the band gap energy diagram including **Arckiy** is appropriately placed for the electron flow inducing recombination and electroluminescence (Figure 14). This produced luminescence exhibits a maximum at ~390 nm, which is blue-shifted in comparison with the lowest energy absorption band at λ_abs_ of 534 nm (Figure 14). This phenomenon is somewhat reminiscent of the S_2_→S_0_ (Soret) fluorescence discussed above for betanin [18]. Computations also indicate that the HOMO and LUMO are, respectively, centered on the apiosyl and crotonic groups, which upon electron recombination creates charge-transfer excited states. Such chromophores would create absorption and luminescence band in the UV-side of the spectrum. It is noteworthy that the geometry used for the calculations exhibits a severely twisted geometry of the chromophore, thus blocking the π-resonance structure. Moreover, it is interesting to note that betanin is practically optically silent in this type of device while the luminance of the Achkiy-containing device is 4.8 Cd m^−2^ when bias voltage of 16.5 V and a current of 34.1 mA are applied. At optimum voltage, the OLED devices produce an irradiance of ~113.3 μW m^−2^. Nevertheless, the “claim for fame” resides in the fact that this natural pigment produces a blue light, a color that is difficult to achieve with this technology.

## 9. Photosensitization of Singlet Oxygen

The direct photoexcitation of molecular dioxygen; ^3^O_2_(^3^Σ_g_^−^) + light → ^1^O_2_(^1^Δ_g_) is an inefficient process as it is a forbidden process by orbital angular momentum projection, by spin, and by “gerade–gerade” selection rules. The use of a photosensitizer (PS) is a necessary alternative (PS + light → PS*; PS* + ^3^O_2_(^3^Σ_g_^−^) → PS + ^1^O_2_(^1^Δ_g_)) where the quantum yield of formation, Φ_Δ_, is (number ^1^O_2_ produced)/(number of photons absorbed by PS). Its evaluation is often performed using a photo-oxidizable organic compound with a known Φ_Δ_ value as a comparison standard [12,91]. The bimolecular photosensitization relies on donor–acceptor collisions, so the lifetimes of excited-state species determine the photo-generated ^1^O_2_(^1^Δ_g_) quantity. In brief, the longer-lived the excited state is, the larger the photosensitization probability. Because the triplet state lives significantly longer than that of the singlet state, the triplet species are more inclined to photosensitization. The singlet state can also photosensitize ^1^O_2_, but its shorter lifetime renders its contribution to Φ_Δ_ somewhat small.

Betalin can also act as a PS of ^1^O_2_(^1^Δ_g_) as previously mentioned with potential applications in dentistry (patent WO2016106173 A1 30 June 2016) and photodynamic therapy (Section 10). But a number of challenges stand out. The most important one is certainly the absence of triplet intersystem crossing permitting to populate the triplet state of betanin based on fs-transient absorption spectrocopy [32,38]. Second, the particularly short-lived singlet excited state (6.7 ps in water) reduces significantly the probability of photosensitization. Knowing that the lifetime of betanin in its S_1_ state can be increased (i.e., to 27 ps), that one uses a viscous solvent (ethylene glycol) conditionally [31,32], this change leads to a parallel drawback that a larger medium viscosity also decreases the number of collisions between the PS* and ^3^O_2_.

This situation then leads to a paradox: betanin may photosensitize ^1^O_2_ as indirectly demonstrated, but the literature proves this natural dye reacts with ^1^O_2_ efficiently [12,13]. The bimolecular quenching of the ^1^O_2_ phosphorescence by betanin occurs with a rate of 1.20 ± 0.15 × 10^8^ M^−1^ s^−1^ in D_2_O, which is near the medium diffusion limit [12]. The use of D_2_O is motivated by the longer phosphorescence lifetime of ^1^O_2_ in this solvent in comparison with water (*τ*_phos_ is 3.57 μs for H_2_O and 77.8 μs for D_2_O at 5 °C; ~3.5 μs and ~67 μs, respectively, at room temperature) [92,93]. The nature of the photoproduct was identified by a combination of UV-vis spectroscopy and mass spectrometry as 2-decarboxy-2,3-dehydrobetanin. The proposed mechanism is presented below (Section 13). Despite the fact that the photosensitivity is amplified when oxygen is in the photo-irradiated solution [94], the oxidation of betanin by ^1^O_2_ does not generate stable peroxides or epoxides, a process that is common for β-carotene [95]. It is noteworthy that isobetanin behaves in the same way as betanin.

## 10. Photodynamic Therapy

The use of betanin as PS in PDT purposes (cancer treatment and antimicrobial) is rather recent (i.e., past 2 years) [11,96]. Despite the existence of commercial cancer PDT drugs, the motivation to expand the medical toolbox is to explore natural substances for sustainability (prices and non-toxicity). In the previous sections, betanin in its singlet excited state is found to be particularly short-lived due to molecular motions and *cis-trans* isomerization. In addition, its thermal stability is challenged over long periods of time in water, and even more so in the presence of oxygen and light. Conversely in the solid state, betanin-based devices such as DSSC and OLED were fabricated and showed activities. In other words, betanin can be rendered more stable and its S_1_ state longer-lived in constrained and highly viscous media.

In the biological world, liposomes are known to encapsulate substances for drug delivery systems. Their microscopic spherical structure is composed of phospholipid bilayers permitting to contain both hydrophilic and hydrophobic substances, and their dimensions can be modulated to obtain small or large vesicles. Thus, selection and optimization is possible and can provide a constrained environment for the guest molecule.

This approach was used for PDT testing with betanin [11]. Indeed, both beetroot juice extract (BRJ) and pure betanin (Bet) can be encapsuled inside liposomes (Lip). From computer simulations (docking), the binding energy of betanin with the liposome is found to be −40.94 kcal/mol, which is rather strong. Using the nitrosodimethylaniline (RNO)/imidazole method, both formulations Lip-BRJ and Lip-Bet photosensitize ^1^O_2_ with yields of, respectively, 12% and 9% after 45 min of irradiation (λ_exc_ = 535 nm). For the PDT experiments, all four possible PSs, BRJ, betanin, Lip-BRJ, and Lip-Bet, were tested against embryonic kidney cell line (HEK 293) and human lung cancer (A549) cell line. The latter two PS composites present the best results and that the photo-induced cytotoxicity is significantly augmented compared to the dark. Under illumination, it is found that the photo-cytotoxicity tests of Lip-Bet on HEK 293 cells and A549 cells exhibit 13% cell (dose = 2000 μg/mL) and 20% cell viability (dose = 1000 μg/mL). Concurrently, lower concentrations are needed to observe significant PDT effects on both cell lines.

The liposome formulation approach was extended to a mixture of betanin and IR806 dye (Bet-IR806-LPs) for codelivery and for targeting two issues: triple-negative breast cancer (TNBC) and a diminished immune system in cancer patients [96]. For the latter purpose, the model bacteria Staphylococcus aureus (*S. aureus*) was selected. IR806 is a near-IR dye (λ_abs_ = 820 nm in water) capable of fluorescence in the near-IR region (λ_abs_ = 840 nm in water) [97], enabling in vivo imaging, including tumor localization. Moreover, IR 806 is also well-known for photothermal therapy [98,99,100]. Altogether, this Bet-IR806-LPs formulation promotes both efficient photothermal (PTT) conversion as well as PDT under near-infrared illumination (λ_exc_ = 808 nm). Indeed, the temperature of the samples rose from 34.7 °C to 51.46 °C and kept a stable plateau of 50.93 °C upon cycling. Cytotoxicity studies on 4T1 TNBC cells using Bet-IR806-LPs under near-IR illumination showed efficient anti-proliferative and anti-migratory properties. Concurrently, the antibacterial spot and colony assays against *S. aureus* also indicated efficient antimicrobial activity for which the Half Maximal Inhibitory Concentration (IC_50_) is 5 μg mL^−1^ under laser irradiation. The biocompatibility of Bet-IR806-LPs was also assessed in fibroblast cell models, thus stressing the importance of nano-sized liposomes for transport in biological media, “guess protection” and subsequent applications.

## 11. Heterogeneous Photocatalysis

Upon application of a cocktail containing beetroot extract onto TiO_2_ NPs (average size ~12 nm; TEM), surface-modified NPs can be prepared and are reminiscent of that built during the construction of DSSCs (Section 7). In a recent application, the photocatalytic degradation of methylene blue dye, as a model for pollution, was performed using such nanocomposites under sunlight radiation [101]. This catalytic degradation showed that 91.41% of methylene blue decomposes over a period of 195 min, and that the kinetic analysis reveals a pseudo-first-order decay (*R*^2^ value of 0.99). Interestingly, after three recycles, the photo-efficiency remains the same.

These same NPs, when subjected to antibacterial assays against gram-neg. bacterial strains (here *E. coli* and Pseudomonas aeruginosa), showed only moderate photoactivity. Concurrently, these NPs exhibit no photoactivity against gram-pos. bacterial strains. This latter result contrasts drastically with the results reported for Bet-IR806-LPs above [96]. It is unclear whether the different illumination conditions used in these two studies (sunlight vs. near-IR laser) play a role in this unexpected outcome [96,101].

**Figure 15:**
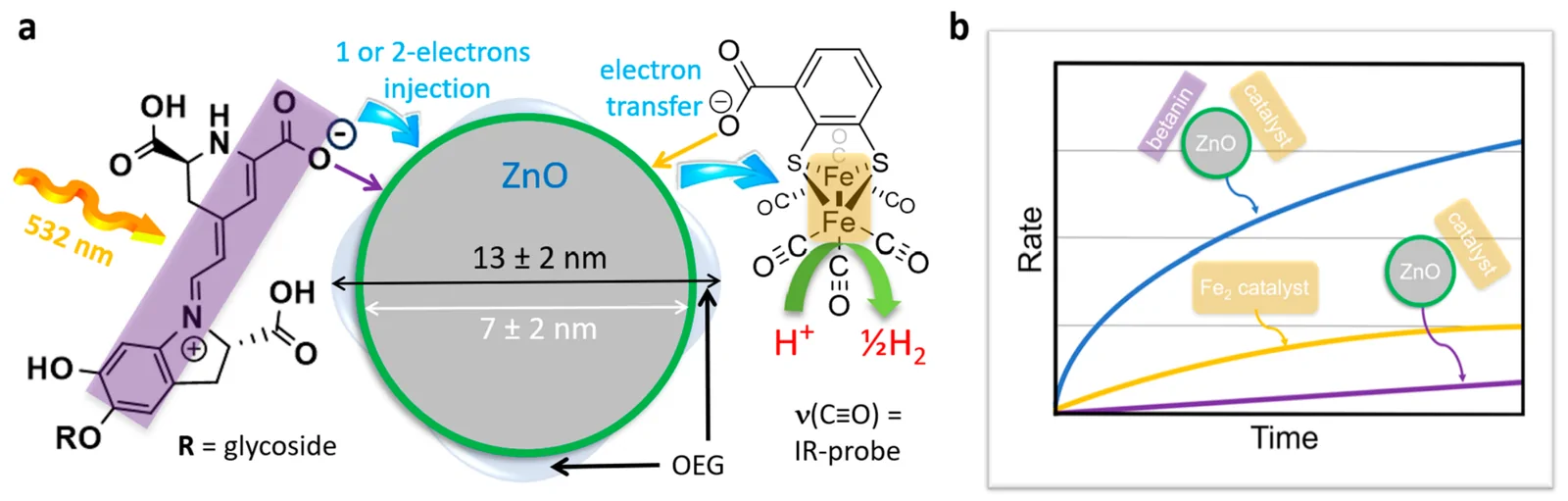
(**a**) Illustration of the concept used to operate the photocatalytic H_2_-evolution reaction. (**b**) Qualitative scheme showing the trend of rate of H_2_-evolution photocatalyzed by ZnO–OEG–B–Cat NPs (blue line), the [Fe_2_(benzenedithiolate)(CO)_6_] catalyst alone (yellow line), and the catalyst linked to ZnO–OEG by a carboxylate (purple line). The total time is 4 h, and each division represents 2 mmol of H_2_ per min.

The use of betanin as antenna and electron donor for injection was also performed, which proved to be determinant in the overall efficiency in the photocatalytic H_2_-evolution reaction occurring at the surface of functionalized ZnO NPs (using carboxylate oligoethylene glycol, OEG as a stabilizing agent) [102]. With an accompanying catalyst η_2_-μ-(benzenedithiolate)-hexacarbonyldiiron(I), [Fe_2_(benzenedithiolate)(CO)_6_], anchored onto the Zn NPs by a carboxylate group, a sacrificial proton donor, trifluoroacetic acid, and a sacrificial water-soluble electron donor, (NH_4_)_2_[Co(H_2_O)_5_(β-HAla)]_2_[V_10_O_28_]·4H_2_O (β-HAla = β-alanine), a photocatalytic system for H_2_-evolution without using a noble metal was also conceived. The principle of action is provided in Figure 15a. Betanin exhibits a large absorption coefficient at 535 nm (65,000 M^−1^cm^−1^) [21,22], and is capable of photo-induced electron transfer (of 1 or 2-electron injection) occurring in less than 20 ps in the semi-conductor ZnO NPs [55]. Based on kinetic traces of the IR peak at 1897 cm^−1^, the ZnO NPs transfer an electron to the catalyst within 5 ns or so. Upon this one-electron transfer to the catalyst, two ν(CO) signals (2074 and 2036 cm^−1^) shift to lower values by 25–50 cm^−1^, which are in line with an increase in electron density on the two iron atoms (increased back donation). This shift is observable in the short μs timescale (0.4 to 1.6 μs) in the time-resolved IR spectra. In the absence of betanin, this catalyst is far less efficient (light blue line vs. black line in Figure 15b).

The use of betanin as a component to increase the efficacity of the photocatalytic H_2_-evolution has also been applied to other NPs [103]. In this case, Bts-Ag NP-linker-TiO_2_-Ru NPs were assembled as photocatalysts, where the Bts-Ag NP moiety is the electron donor, and TiO_2_–Ru NP is the electron acceptor. A series of betalains (betanin, neobetanin, betalamic acid and cyclo-DOPA 5-*O*-glucoside (cDOPA)) are used as *regenerators* and has the capacity of retarding electron/hole recombination. The semi-conductor TiO_2_ has the property to increase both the distance and duration charge/hole separation. The ruthenium NPs are a common catalyst for H_2_-evolution, and 4-aminobenzoic acid links the Ag NPs and TiO_2_ NPs. This linker is well-known for its excellent π-electronic communication and coupling. Finally, the Ag NPs (monodispersed sphere of 28–30 nm; λ_abs_ = 405 nm) can also act as light absorbers, as well as the betalains. The sacrificial electron donor is methylene blue dye, whereas water is the proton source. Using a 405 nm CW laser, the photocatalytic study revealed that the solar-to-fuel efficiency (quantum yield) is found to be 19.9 ± 0.5%, which is considered large even nowadays. It is noteworthy that it is assumed that each absorbed photon to a single electron transfer in this estimation. The dynamic study of the photophysical processes (monitored by ultrafast transient IR spectroscopy at 2081 cm^−1^) indicates that the linker (4-aminobenzoic acid) contributes to increase the electron transfer rate from Bts-Ag NP to TiO_2_ by three- to four-folds as witnessed by a rise time of 600–700 fs (pulse width = 200 fs). This electron injection is completed after 1.5 ps. Between the rise time recombination processes quasi-unavoidably occurs with time dynamics of τ_1_ = 5 ps (93%) and τ_2_ = 80 ps (7%). These time values are conveniently slower than those observed in the betanin-ZnO NPs (Figure 13; 1.5 and 20.6 ps). Finally, the charge separation is estimated to be longer than 5 ns, which is long enough for the reduction in water.

Other photocatalytic systems have been reported using betanin-containing extract from dragon fruit (*Hylocereus polyrhizus*) and ZnO NPs, but the thermal treatment of the biowaste (70–80 °C) provokes a change in the red color of the initial residue to yellow [104]. The significant color change unambiguously indicates that the isolated yellow solid mixture does not contain betanin anymore. Nevertheless, the photodegradation of methylene blue was also observed and investigated. This outcome suggests that in a cocktail of betalains, betanin is certainly not the only contributor to the overall antenna effect and electron injection in the NPs and, thus, to the overall photocatalysis. An individual quantification of the photocatalytic performance for each molecular component in such cocktails (beetroot, etc.) as well as the synergic effect provided by singlet–singlet energy transfer between two different betalains are worth further investigation.

## 12. Photo-Instability

**Figure 16:**
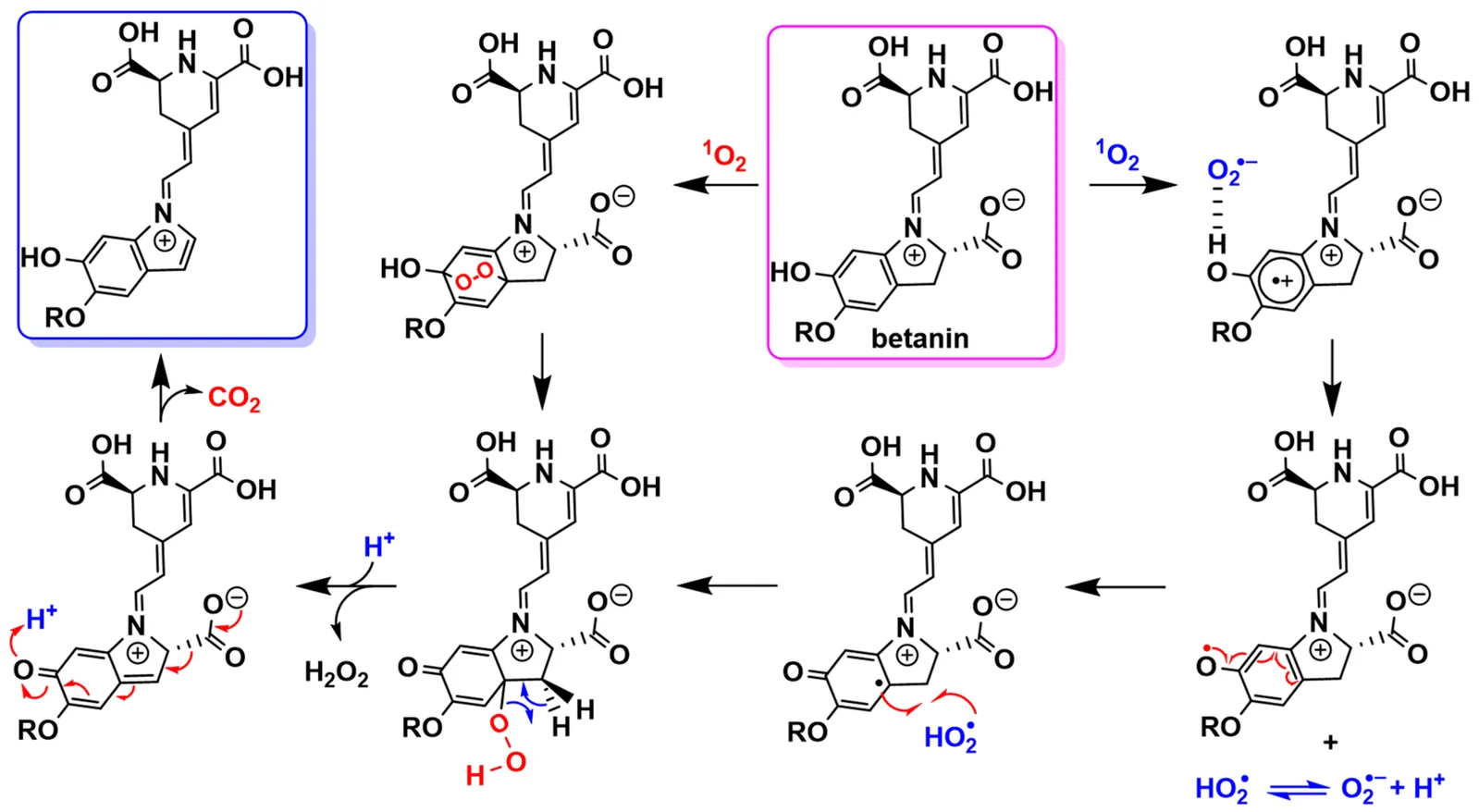
Proposed mechanism of the rapid photodegradation of betanin (purple box; R = glucoside) in water in the presence of oxygen into 2-decarboxy-2,3-dehydrobetanin (blue box) according to [12].

Betanin photodegrades relatively rapidly when it is illuminated in aqueous solution in the presence of oxygen (via the formation of the ROS type II; singlet oxygen), as reported multiple times [12,14,105,106]. 2-decarboxy-2,3-dehydrobetanin is the main observable and isolatable photoproduct, and a proposed mechanism has been provided (Figure 16) [12]. This situation creates a paradox since betanin photosensitizes singlet oxygen (Section 9 and Section 10), but at the same time, its antioxidant properties make this natural pigment prone to capture this ROS that it just generated with the presence of light. Consequently, the photogeneration of ^1^O_2_ creates a photo-oxidative stress on plants (and inside poorly degassed devices), which is relieved by the suicidal mission to capture it afterward. Thus, this paradox gives betanin a dual opposite functionality, which ultimately reduces their individual performances. In the proposed mechanism, the presence of a phenol group was positioned in a way such that decarboxylation becomes easy in the presence of a protic media through π-conjugation. A parallel study, mainly aimed at soft drinks, on the effect of UVB irradiation (λ_exc_ = 300 ± 50 nm; 4.7 ± 0.3 mW/cm^2^; 25 °C) on the stability of betanin (in an N_2_-purged aqueous solution for 5 min), which were monitored by UV-vis spectroscopy, shows that the photodegradation follows a biphasic first kinetic of 0.228 h^−1^ (at the beginning) and 0.35 h^−1^ (at the end), meaning that the stoichiometry is preserved, as evidenced by the presence of isosbestic points in the spectra. Thus, the presence of a multi-step degradation process under these conditions is concluded [105]. While full photodecomposition is observed within 6 h, in the presence of air, total photodecomposition is observed within the time necessary to measure one spectrum (mins).

It is also well-known that the instability of betanin decreases markedly with the temperature, where light has little effect at 55 °C [14]. At high temperature, the thermal degradation has a greater effect than light irradiation, where again, the presence of oxygen accelerates the photodecomposition. However, to the best of our knowledge, no study was performed to identify all photodegradation products, notably when solution turns colorless. Concurrently, the thermal products have been identified and presented in a review [94]. The list of products includes: neobetanin, 15-decarboxybetanin, 17-decarboxybetanine and isobetanin, isobetanin is typically the first one formed, and then the colorless cyclo-Dopa-5-O-b-glycoside, and the yellow betalamic acid and isobetalamic acids (note that the position numbering changes from one investigation to another). Similarly, the oxidation products when betanin is subjected to a one-electron oxidation (by 2,2′-azinobis(3-ethylbenzthiazoline-6-sulfonic acid)), have also been identified: 2,17-bidecarboxy-2,3-dehydrobetanin, 2-decarboxy-2,3-dehydrobetanin, 2,15,17-tridecarboxy-2,3-dehydroneo-betanin, 2,17-bidecarboxy-2,3-dehydroneobetanin, and 2-decarboxy-2,3-dehydroneo-betanin, as determined by HPLC [107]. There is a possibility that several of these products and intermediates are the same during both thermal and photochemical degradation of betanin. It is noteworthy that the stability of betanin decreases when in the presence of water, which is consistent with the proposed mechanism of Figure 16 as being a proton source [108].

## 13. Nonlinear Optical Materials

**Figure 17:**
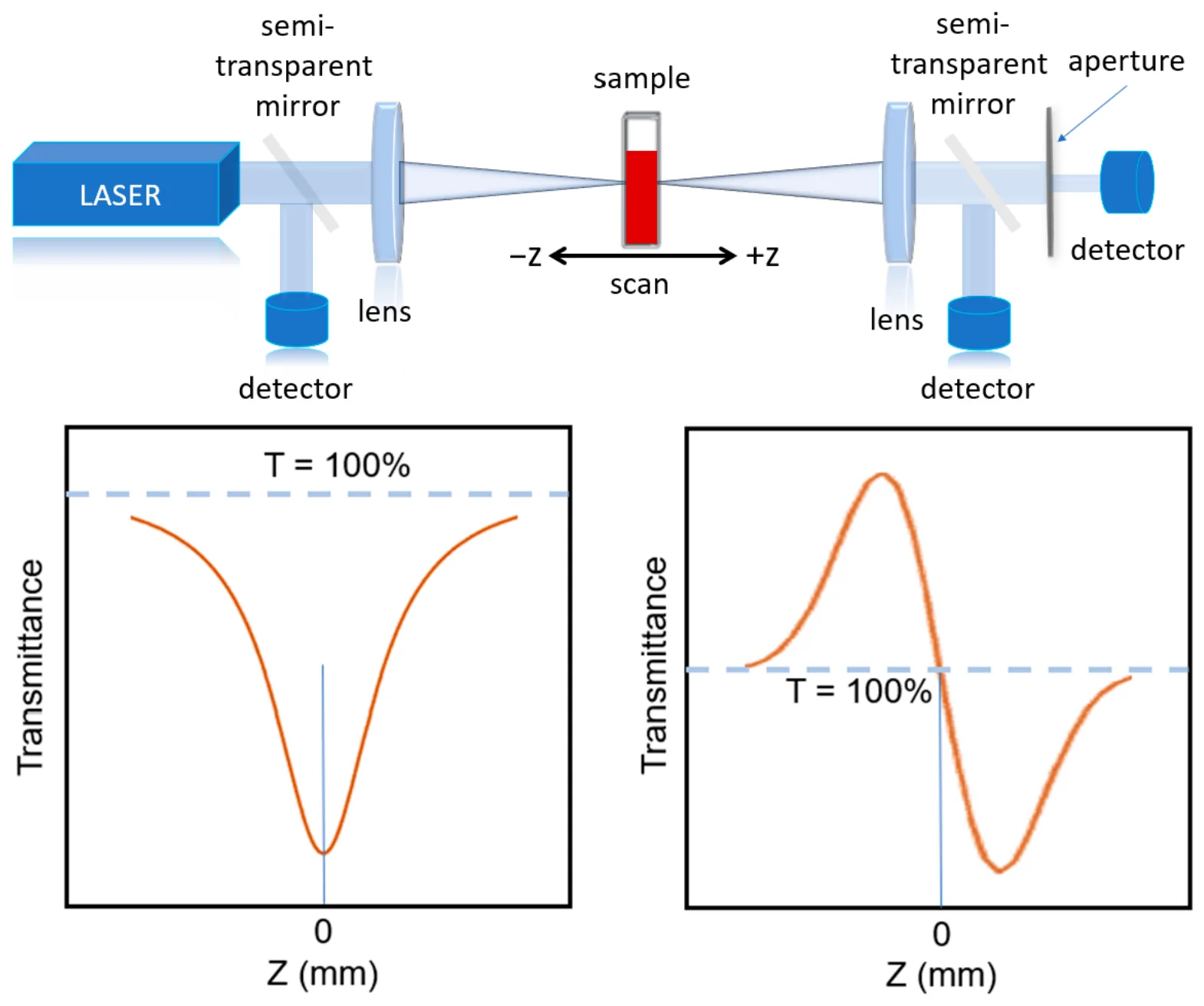
**Top**: scheme illustrating the apparatus set-up for Z-scan measurements. Other small variations in set-ups exist. The first detector after the laser source is to correct the signal due to intensity variation. The typical Z-scan span ± 2 cm. **Bottom left**: Typical open-aperture Z-scan curve (the back detector is used after the aperture). For betanin, the minimum value is 58%. **Bottom right**: Typical pure nonlinear refraction curve. For betanin, a typical light source is a low-power CW diode laser at 532 nm because of the match between this λ_exc_ and the absorption maximum of the chromophore (535 nm). For betanin, the maximum and minimum transmittance values are 160 and 40%, respectively.

Two-photon absorption spectroscopy, such as pump–delay–probe experiments (UV-vis and IR), provides dynamic information on the molecular excited states, on plasmonic, and on charge-separated states, etc. Concurrently, under intense laser excitation, two-photon absorption (no delay) under an intense field by a material is also possible. This phenomenon leads to nonlinear optical phenomena, and betanin in solution and various matrices has also been the subject of experimental and computational investigations [30,109,110,111,112]. When two photons are absorbed at the same time by a molecule, the Beer–Lambert law no longer applies, resulting in a lack of detected transmitted photons by the back detector. The most popular set-up to extract NLO information is called the open and closed aperture Z-scans (Figure 17: top). Using a lens, a focal point exists at a given distance where the laser flux is optimum, and the nonlinear effect is maximum (less photons transmitted), leading to a V-shaped curve (Figure 17: bottom left). The two main parameters sought are the nonlinear refractive index (optical Kerr effect), *n*_2_, and the nonlinear absorption coefficient β.

A typical experiment consists of using a solvent medium (using an appropriate methodology to compensate for the nonlinear behavior of the solvent), using a 1 cm cuvette. The sought parameters become both solvent- and concentration-dependent. The β-values for betanin mixed in a 1.6 vol.% with a given solvent are 2.903 × 10^−10^ (water), 1.655 × 10^−10^ (ethanol) and 0.981 × 10^−10^ M/W (methanol); λ_exc_ = 532 nm, Q-switched Nd-YAG laser; 10 Hz; 7 ns pulse length; fluence of 436 MW/cm^2^), which follows the polarity index of the solvent (water = 9; ethanol = 5.2; methanol = 5.0) [110]. These variations are attributed to solute–solvent interactions. A computational study provided calculated data but they turned out to be off by many orders on magnitude [30].

Betanin has also been employed as a doping agent to improve the NLO properties of ZnO nanoplates embedded in polymer matrices, such as polyvinyl alcohol (PVA) [111,112]. This improvement stems from a photo-induced electron transfer due to the electron rich properties of betanin. A clear demonstration stems from the influence of the laser intensity versus the NLO outcome. Indeed, the laser intensity can also alter these properties in betanin/ZnO nanoplates inside polymer matrices [111,112]. In earlier studies, this demonstration was performed, but there was no mention of parallel studies on the ubiquitous presence of the photo-induced charge separations (betanin*-ZnO → betanin^+^^•^ + [ZnO]**^−^**^•^) as discussed above (see Section 6 and Section 7, and Section 11).

***General Discussion***

## 14. Discussion and Perspectives

Betanin exhibits three paradoxes concerning its excited-state properties and applications. The first one arises from the ultrafast internal conversion rate where over 99% of the absorbed light energy is dissipated under the form of heat, and yet, a short-lived fluorescence (ps-timescale) is still observed, and photo-induced electron transfer as well as the design of DSSCs (with modest power photoconversions; PCE ≤ 4%) were reported. The second paradox stems from the absence of intersystem crossing (S_1_~~>T_1_), and yet PDT (with singlet oxygen) was performed. The third one is while betanin is inclined at photosynthesizing ^1^O_2_, its antioxidant behavior is also prone to trap this same ROS in a sacrificial manner. These three observations stem from one fact, the excited-state dynamics. When betanin is subjected to different experimental conditions such as solvent, viscosity, presence or absence of oxygen, solid state vs. solution, encapsulated into a host or not, or different center-to-center distances with dyads in bimolecular processes, the non-radiative rate constants are bound to vary. Just a subtle variation is enough to induce observable changes, even modestly, and the relative population, following a given photo-induced process, varies. In addition, the conclusion is that there is no intersystem crossing occurring in betanin as assessed by fs-transient absorption spectroscopy and cannot be rigorously taken as a rigid fact. The firm and reproducible intensity of a very weak signal cannot be necessarily detected with this technique. A very weak fraction of the excited-state population may exist but not be large enough to be detected by laser-transient absorption spectroscopy. On the other hand, the conclusions drawn from PDT experiments on cell lines are unambiguous.

In attempts to improve this situation, various convenient solutions exist. The first one consists of post-functionalization of the extracted betanin as recently suggested and aiming at improving DSSC performances [113]. This solution would move the final product, prior to application, from a natural, low-cost and abundant source to a bio-sourced product that uses an extra, and not necessarily environmentally friendly synthetic step to obtain it.

One possible explanation is that any charge-transfer admixture (betanin^+•**…**1^O_2_**^−^**^•^) to the triplet state wavefunction in the betanin**^…^**^3^O_2_ collision initiates a spin–orbit coupling contribution and intersystem crossing [113,114]. Thus, this triggering radical pair is prone to enhance the triplet state population of betanin in the medium and promotes further photosensitization of ^1^O_2_ and betanin^+•**…**1^O_2_**^−^**^•^ radical pair (chain reaction). Consequently, the reported PDT processes [11,96] may then operate through both ^1^O_2_ and the superoxide ^1^O_2_**^−^**^•^ and betanin serves as a sacrificial electron donor. No experiment for the detection of radicals was performed in these works (radical scavengers). So, this explanation is hypothetical.

In light of past investigations, two favorable conditions led to applications (DSSCs, OLEDs and PDT). The first one is solid state and high viscosity. It was well-argued that upon increasing the quantity of betanin at the surface of TiO_2_ NPs, the inter-dye distance decreases to the point where the π-stacked betanin generates a broader absorption band thus harvesting more photons, which contributed to increasing the PCE (3%; a record at that time) [75]. The close stacking also provides some steric to minimize molecular motions and physical protection against potential reactants, inducing betanin degradation. The second condition is the encapsulation of betanin. In this review, liposome encapsulation was used for drug delivery and provided “protection” to betanin against degradation until photosensitization (Section 10). The concept of encapsulation of betanin have been recently applied using hydrocolloids and showed to increase the thermal stability up to 80 °C [114]. However, the most impressive photophysical behavior is shown in Figure 10, where spectrum is the most red-shifted and exhibited a high intensity [46]. The “recipe” for such an intensity arises from the beetroot cocktail itself where the antenna effect from the other betalains present just slightly blue-shifted absorption bands in a way the J-integral of Equations (1) and (4) are optimized (Section 5). This idea was also applied in DSSC technology where betalain-containing NPs currently has the record PCE [83].

Key experiments often encountered in heterogeneous photocatalysis and cytotoxicity involving ROS have seemingly not been performed. The proposed mechanism of Figure 16, where the photodecomposition of betanin in the presence of oxygen and water is presented, involves both types of ROS. It would be very informative to systematically perform tests with specific radical scavengers, spin traps using electron paramagnetic spectroscopy, and ^1^O_2_ traps to identify the active ROS in each situation.

Betanin in its triplet state has not been explored, and still PDT has been observed. In fact, the characterization of betanin* should be easily accessible via computations or cryogenic spectroscopic techniques in glassy matrices or in the solid state (no cis, trans-isomerization and photo-induced oxidation are possible). Moreover, heavy atom effect, which increases spin–orbit coupling and consequently the rate of intersystem crossing, can also be exploited by post-functionalization. For example, the bromination or iodation of natural betanin should be considered to improve PDT performance. A classic example is BODIPY (4,4-difluoro-4-bora-3a,4a-diaza-s-indacene) for which the incorporation of a heavy atom such as Br and I onto the inert non-halogenated BODIPY PS improves drastically the PS ability of this chromophore going from negligible to very performant. For betanin, its abundance, low price, and non-toxicity represent a good opportunity for the development of bio-sourced new PS [115,116].

The NLO section is divided into two main topics: (1) betanin in solution, and (2) betanin used as doping agent to improve the NLO properties of ZnO nanoparticles in polymer matrices. With the conclusions drawn throughout this review that (1) the medium rigidity improves the photophysical properties (i.e., fluorescence intensity and excited-state lifetime), and (2) betanin photodegrades when irradiated with visible light in the presence of oxygen, it now appears beneficial that betanin-based NLO materials would be best fabricated under inert atmosphere and the device sealed from air (the same way as organic solar cells are).

## 15. Conclusions

Betanin is an underestimated and underexploited natural pigment for applications, when under visible light irradiation, mainly due to its thermal and photochemical “fragility” in the presence of water and oxygen as well as its intrinsic ultrafast rate of internal conversion, including *cis-trans* isomerization, coupled with a seemingly negligible rate of intersystem crossing. In addition, the possibility that radical ion pairs are formed during illumination can also contribute to the betanin instability (and the PDT properties).

Recent developments demonstrated that both the solid state and the encapsulation approaches offer a partial “protection” to betanin in its excited state, as well as increasing the excited-state lifetimes. Fortunately, technologies such as DSSC, OLED and PDT have been developed within the past decade or so and offer clues on how to go about these challenges. Rigorous anaerobic conditions are therefore needed to fabricate photonic devices based on betanin (such as organic solar cells, DSSC, OLED, NLO materials) exhibiting optimized properties and performances as well as device rigidity when possible. Essentially, there is still room for improvement, both at the engineering and molecular levels. However, the reported performances are modest compared to the state-of-the art (DSSCs and NLO materials), or simply in its infancy (OLED).

Finally, for sustainable development, the heterogeneous catalytic generation of solar fuels (H_2_, CH_4_) presented in Section 11 represents the most promising technology to be developed. Indeed, the ingredients to fabricate the photocatalysts are both readily available and inexpensive, and hence, easily replaceable (betanin, ZnO, and iron complexes).

## Figures and Tables

**Table 1:** List of investigations on betanin-containing DSSCs.

NP	Dye or Mixture of Dyes	Best η (%)	[Ref]	Year
TiO_2_	*Opuntia soehrensii* (**AY1**; **AY1**+citric acid)	4	[83]	2024
TiO_2_	betacyanin:curcumin:chlorophyll 2:1:1	3.57	[76]	2024
WO_3_	betanin	3.52	[68]	2021
TiO_2_/ZnO	betanin	3.25	[64]	2023
TiO_2_	betanin aggregate	3.0	[75]	2016
TiO_2_	betanin	2.7	[27]	2011
TiO_2_	*Beta vulgaris rubra* (Red beetroot) extract	1.75	[66]	2009
ZnO/Au	betanin	1.71	[74]	2015
IO-TiO_2_	betanin (IO = inverse opal)	1.60	[80]	2026
Al@TiO_2_	Red beetroot extract	1.5	[72]	2019
TiO_2_	wild Sicilian prickly pear fruit juice extract	1.26	[67]	2010
Ag	betanin/lawsone	1.02	[21]	2020
TiO_2_	betanin/betaxanthins	0.82	[69]	2020
TiO_2_	betanin/lawsone	0.793	[71]	2019
TiO_2_	betalains	0.69	[79]	2014
TiO_2_	*Amaranthus caudatus* extract	0.61	[73]	2015
TiO_2_	*Bougainvillea spectabilis* and *B. glabra*	0.48	[86]	2011
TiO_2_	betanin	0.45	[81]	2025
ZnO/Au	betalains	0.4	[50]	2012
TiO_2_	betanin; betanin plus CdS	0.37	[77]	2024
TiO_2_	betanin	0.30	[65]	2024
ZnO	chlorophyll plus betalains	0.294	[78]	2016
ZnO	Red beetroot extract	0.28	[85]	2011
TiO_2_	betanin	0.217	[82]	2023
{111}-TiO_2_	Red beetroot	0.13 < η < 0.50	[70]	2022
TiO_2_	betalains	0.07	[84]	2008

## Data Availability

No new data were created or analyzed in this study.
